# Time and video speed perception: a comprehensive investigation of the relation between estimated video speed, clip duration and original duration

**DOI:** 10.1186/s41235-025-00637-2

**Published:** 2025-07-01

**Authors:** Verena Steinhof, Anna Schroeger, Roman Liepelt, Laura Sperl

**Affiliations:** 1 Department of General Psychology: Judgment, Decision Making, Action, Faculty of Psychology, University of Hagen (FernUniversität in Hagen), Universitätsstraße 37, 58097 Hagen, North Rhine-Westphalia Germany; 2 Department of Experimental Psychology, Faculty of Psychology, University of Gießen (Justus-Liebig-Universität Gießen), Alter Steinbacher Weg 38, 35394 Giessen, Hesse Germany; 3 Center for Mind, Brain and Behavior, University of Marburg, Justus Liebig University Giessen and University Darmstadt, Hans-Meerwein-Straße 6, 35032 Marburg, Hesse Germany

**Keywords:** Video speed, Slow motion, Time lapse, Clip duration, Original duration, Time perception, Verbal estimation, Time reproduction task

## Abstract

While decades of research have deepened our understanding of time perception, the perception of (manipulated) video speed has been relatively underexplored but is gaining interest with recent technological advances. This study systematically investigated human perception of *video speed*, *clip duration* and *original duration* across slow motion, original speed and time lapse. Results showed that participants consistently underestimated video speed in time lapse and overestimated it in slow motion, suggesting a tendency toward an internal perceptual standard. A similar pattern emerged for clip duration with videos being generally overestimated in their duration when played in accelerated speed, and underestimated in slow motion. For original duration estimations, this pattern was reversed. Surprisingly, while estimations of clip and original duration were highly correlated in all video speed conditions, both measures were uncorrelated with estimated video speed. However, an exploratory analysis suggested that the distorted perception of original duration may, at least in parts, still be rooted in participants relying on their own (but biased) interpretations of clip duration and video speed. Most importantly, these results reveal distortions in perception caused by altered video speeds, suggesting caution when employing these video techniques for judgments and decision-making. The findings provide foundation for further research, investigating the cognitive mechanisms of human video speed perception.

**Supplementary Information:**

The online version contains supplementary material available at 10.1186/s41235-025-00637-2.

## Significance

In today’s digital era, videos have become not only a source of entertainment but also a key medium for communication and information sharing. Video speed manipulations, such as slow motion and time lapse, are widely used across various contexts. While slow motion is commonly employed to enhance observational clarity by highlighting details that are not visible at original speed, recent studies have shown that such manipulations can introduce perceptual biases, distorting judgments and decisionmaking. This is especially important when videos are used as evidence, such as in sports or legal proceedings. Research indicates that humans have difficulties to accurately perceive altered video speeds. Inspired by previous research, our study aimed to take a step back and to investigate the fundamental mechanisms underlying human perception of altered video speed. The results uncovered inaccuracies in both speed and duration estimates, suggesting a complex, non-linear relationship between these variables alongside individual differences. Thereby, this study is one of the first systematic investigations of the key factors that influence human video speed perception. It underscores the need to be aware of how speed-altered videos can affect perception and decision-making in real-world applications and aims to provide a starting point for future research in this emerging field.

## Introduction

In the digital era, the possibility to alter video speed with a simple click has revolutionized how temporal processes are displayed and experienced. Techniques like slow motion or time lapse are now easily accessible through smartphones and computers. While often used for cinematic effects, video speed manipulation also serves documentary purposes, making fast or slow changes more perceptible. Slow motion reveals details missed at original speed, enabling detailed analysis (e.g., determining whether a ball is in play during a sports match), while time lapse compresses long events into short sequences (e.g., tracking months of construction progress). These techniques are widely applied in fields such as sports (Spitz et al., 2017), education (Davis et al., 2021; Murphy et al., 2022), and criminal investigations (Caruso et al., 2016).

### Video speed perception

Despite the widespread use of video speed manipulation, research on human perception of altered video speeds remains surprisingly limited, with only a few studies examining this in real-life contexts (de’Sperati & Baud Bovy, 2017; de'Sperati et al., 2021; Rossi et al., 2018; Zuliani et al., 2019; Mather et al., 2017). Initial findings indicate that humans struggle to perceive speed changes accurately (de’Sperati & Baud Bovy, 2017).

For instance, de’Sperati and Baud Bovy (2017) found that participants had difficulty recognizing speed manipulations, showing deviations of up to 12% when estimating original speed compared to time lapse or slow motion. Similarly, Mather et al. (2017) demonstrated that speed estimations are influenced by prior exposure, with judgments shifting toward the speed presented in the adapting video, reflecting an internal standard (see also Grivel et al., 2011).

Rossi et al. (2018) reported a tendency to underestimate video speed (see also de’Sperati et al., 2021; Zuliani et al., 2019), with detection varying based on clip content. For example, participants found it more difficult to detect speed changes in physical scenes, such as water waves, compared to clips combining human and physical motion. Interestingly, clip duration estimates were also underestimated, but they did not correlate with speed estimates, suggesting that they were probably processed independently.

### The interplay between video speed and time perception

The absence of correlation between participants’ estimates of clip duration and video speed, as observed by Rossi et al. (2018), challenges conventional expectations regarding the dynamics of video speed and time. In video editing, altering speed follows simple mathematical rules involving temporal scaling: while the physical original duration of an event remains constant, the clip duration varies proportionally to the acceleration (time lapse) or deceleration (slow motion) factor. Thus, *video speed*, *clip duration*, and *original duration* are key variables in understanding the relationship between speed and time perception in videos. Mathematically, this relationship can be quantified as the product of speed and duration, where original duration is calculated as clip duration multiplied by the video speed factor (e.g., a 5 s clip duration multiplied by a speed factor of 2 results in an original duration of 10 s). Hence, at least from a mathematical perspective, the original duration can be directly derived from the other two variables (clip duration and video speed). However, it still remains unclear whether similar mental operations are performed, at least subconsciously, in order to form an idea of how long an action actually lasted in real time.

In the relatively unexplored field of video speed perception, recent studies have revealed significant effects of slow motion on estimating original duration. When videos are played in slow motion, there is a tendency to overestimate the original duration of actions, i.e., the action is perceived as longer than it actually was. This phenomenon was recently described as overestimation bias (Sperl et al., 2021) and intensifies with greater video slowdown (Schütz et al., 2021; Sperl et al., 2021). This bias in slow motion seems to influence not only time perception but also cognitive evaluative processes, leading observers to erroneously perceive more intentionality in actions due to a misleading perception of greater available planning time (Caruso et al., 2016; Spitz et al., 2018).

Sperl et al. (2021) suggested that this bias was due to underestimating the slow-motion factor (thus perceiving the video as faster than it actually is), rather than overestimating the original duration itself, aligning well with the mathematical formula described above. This formula suggests that if the video speed is overestimated, it directly results in an overestimation of the perceived original duration. This assumption was supported by their novel finding that informing the participants about the slow-motion factor eliminated the overestimation bias. Providing video speed information seemed to correct the misperception of slow-motion intensity and the subsequent inaccurate conversion to original duration. A follow-up study by Hüttner et al. (2023) replicated the overestimation bias in slow motion and its correction with video speed information. However, providing video speed information did not eliminate the increased intentionality attribution.

In contrast, Rossi et al. (2018) observed that speed and duration perception were largely independent. Hence, further research is needed to clarify the cognitive mechanisms that come into play when watching speed-altered video material. Whether factors known to influence time perception, such as age, gender (Block et al., 1999), attention (Knudsen, 2007; Pashler et al., 2001), or stimulus characteristics like speed and motion (Brown, 1995; Kanai et al., 2006; Matthews, 2011), also apply to video speed perception remains an open question. Initial evidence suggests that some of these variables impact video speed perception as well. For instance, older adults (de’Sperati et al., 2021) and young children (Zuliani et al., 2019) tend to underestimate video speed, potentially indicating a U-shaped relationship. This misperception may be linked to compensatory mechanisms associated with motor slowing due to aging. Additionally, factors like clip content (Rossi et al., 2018) and prior stimuli (Mather et al., 2017; Grivel et al., 2011) have been shown to influence performance in tasks related to video speed.

### Measurement methods in video speed and time perception research

Different methods have been employed to assess video speed perception, including verbal estimation (Hüttner et al., 2023), detection tasks (de’Sperati & Baud Bovy, 2017), comparison tasks (Rossi et al., 2018), and speed adjustment tasks (Zuliani et al., 2019; de’Sperati et al., 2021; Rossi et al., 2018). Regarding duration perception, two common methods are verbal estimation (VE) and time reproduction task (TRT) (Bindra & Waksberg, 1956). In VE, participants estimate the perceived duration in time units like seconds, while TRT requires them to reproduce the observed duration, often by pressing a button. However, different measurement methods have been shown to yield varying results, sparking debate about which is more accurate or precise (e.g., Damsma et al., 2021; Yerkes & Urban, 1906; Asaoka & Watanabe, 2015; Spencer, 1921). Some researchers suggest that VE and time (re)production tasks (Thönes & Hecht, 2017) rely on distinct cognitive processes (Clausen, 1950; Danziger & Du Preez, 1963; Kruup, 1961), while others find strong correlations between them, suggesting a shared intrapersonal basis for time estimation (Du Preez, 1963; Asaoka & Watanabe, 2015; Carlson & Feinberg, 1970). The influence of different measurement methods on estimating clip and original durations under varying video speeds remains unresolved. For example, Sperl et al. (2021) suggested a connection between original duration and video speed in slow motion using VE, while Rossi et al. (2018) found no correlation between clip duration and video speed using TRT. Since it remains unclear whether these divergent findings result from using different methods, this study will incorporate both VE and TRT.

### Aim of the study

The aim of this study was to investigate human perception of altered video speeds by examining the interplay between *video speed*, *original duration*, and *clip duration* across different speed conditions. Thereby, we also tested whether those subjective estimations underly the same mathematical conversions as their physical equivalents. Based on prior research (e.g., Hüttner et al., 2023; Sperl et al., 2021, de’Sperati & Baud Bovy, 2017; Rossi et al., 2018), the following hypotheses were derived:

H1) The deviations in video speed, original duration and clip duration estimations, significantly vary across the different video speeds (slow motion, original speed, time lapse).

H2) The estimated video speed, original duration, and clip duration of displayed actions show significant correlations among each other within different speeds (slow motion, original speed, time lapse).

Notably, to examine potential discrepancies between different measurement methods, we compared responses provided by a verbal estimate (VE) with performance in a time reproduction task (TRT) for estimated clip duration and estimated original duration within the different video speeds testing for differences and correlational relationships between these two different measurement methods.

## Method

### Participants

The study included 219 participants recruited via personal outreach, dissemination through social networks, and the virtual online laboratory of FernUniversität Hagen. After excluding four participants (2%) who reported non-serious participation and 38 individuals (17%) who reported technical issues, the final sample size was *N* = 177 participants^1^ (114 females, 63 males, age: *M* = 36.79 years, *SD* = 15.58, range: 19-87). Participants were incentivized with course credits. The study was part of a study series approved by the Ethics Commitee of the Faculty of Psychology of the FernUniversität Hagen (ethics approval no.: EA_482_2022).

### Materials

Three muted video clips, each depicting brief, comparable everyday activities in a consistent setting, were created for this study. All videos were filmed with a Samsung Galaxy S21 FE, in 120 fps and Full HD, from a straight-on angle, 1.5 m away. The videos featured the same female actor, who was seated at a table in front of a white background, wore a black hat to obscure her face, minimizing distractions from facial expressions. In video A (dice), she shakes a dice cup and pours the dice onto the table. In video B (teapot), she pours tea from a teapot into a cup. In video C (match), she repeatedly lights five tea lights, striking a match from a matchbox in her left hand.

During stimulus creation, we aimed to minimize confounding effects on speed perception, such as emotional valence, distracting objects, or large trajectory movements, which could influence participants’ temporal experience (Cohen et al., 1953; Loeffler et al., 2018). Therefore, the selected clips all featured a human performing simple everyday actions, combining human and object motion to provide cues based on real-world physical laws (Rossi et al., 2018). This approach allowed participants to infer the original speed of the movement, unlike stimuli typically used in motion speed paradigms (e.g., geometric shapes), where an original speed per se does not exist. The three selected video contents were chosen to represent approximately natural speeds for these actions.

The original clips were assigned different durations (see Table 1) to introduce variety and prevent monotony. Edited using Adobe Premiere Pro (Adobe, 2023), each video was presented in three playback speeds: original speed at x1.0 (100% video speed), slow motion at $\text {x2.3}^{(-)}$ (43.5% of original speed), and time lapse at $\text {x2.3}^{(+)}$ (230% of original speed)^2^, all at 30 fps.

The entire action was preserved in both time lapse and slow motion, leading to variations in video length. The high recording frame rate of 120 fps ensured smooth playback across all speeds, maintaining a final presentation rate of 30 fps, which meets the standard for natural-looking playback (Nasiri et al., 2015; Pazhoohi & Kingstone, 2021). This resulted in a total of nine video stimuli (see Table 1). The videos are available on OSF (https://osf.io/9asjz/).

**Table 1:** Video clip durations across different video speeds

	Video A (dice)	Video B (teapot)	Video C (match)
Slow motion (x$2.3^{(-)}$)	11.5 s	15 s	16.1 s
Original speed (x1.0)	5 s	6.5 s	7 s
Time lapse (x$2.3^{(+)}$)	2.2 s	2.8 s	3 s

*Note.* The durations for the videos at different speeds are based on their original duration, modified by a factor of 2.3. The content of the videos remains unchanged across all speeds

### Design

The study used a strategic randomization where participants were randomly assigned to one of six conditions, with each participant estimating three combinations, where each of the three video clips (A, B, C) was paired with one of three video speeds (slow motion, original speed, time lapse) (see SI, Table S1 for details).

This approach helped mask the symmetrical video speeds, preventing participants from making calculations based on previous estimates, thus promoting independent estimations across different speeds. Each participant performed 15 estimations, i.e., one estimation for each of the five dependent variables (video speed, clip duration VE, clip duration TRT, original duration VE, original duration TRT) for each video speed (see Sect. Dependent Variables). The participants watched the video clips in randomized order and provided their estimations immediately after viewing each corresponding video.

Importantly, participants were informed before viewing the videos that they would need to make duration and speed estimations throughout the experiment. However, to reduce the use of counting or computational strategies, the specific task for each trial was introduced only after viewing each video.

### Dependent variables

**Fig. 1:**
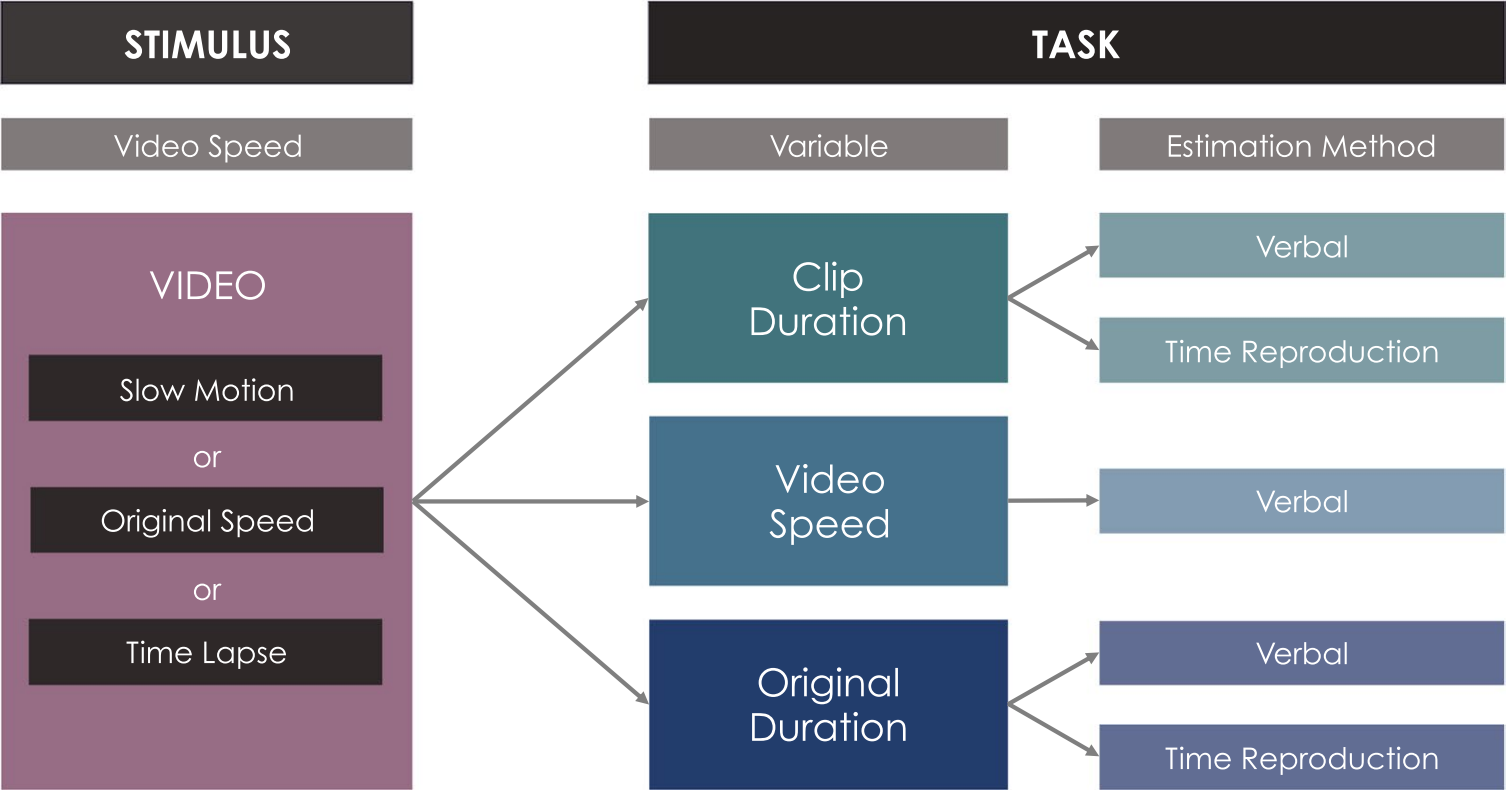
Design overview. In each trial, participants viewed one of three video clips at one of the three randomly assigned video speeds (slow motion, original speed or time lapse). The assignment of video clips to video speeds was predetermined through the randomization method described in Sects. Design and Procedure (see also SI, Table S1). After viewing a video clip, participants were required to provide a single estimation - either for clip duration, video speed, or original duration, using one of the predefined estimation methods. This process was repeated across 15 trials, so that by completion, each participant had provided estimations for all three key variables using all estimation methods (i.e., five estimations for each of the three video clips), in its assigned video speeds

For a variable and design overview, see Fig. 1.

***Video speed***

The measurement of *video speed* required participants to first choose among the options slow motion, original speed or time lapse and then enter a speed factor^3^ up to one decimal place.

***Clip duration***

The *clip duration* refers to the total duration of a video clip and was assessed with VE (entering the duration in seconds up to one decimal place) and TRT (pressing and holding the space bar for the duration matching the perception of the duration of the just-watched video clip).

***Original duration***

The *original duration*^4^ in seconds of a presented scene refers to the real-time duration of the entire scene depicted in the video. Like for *clip duration*, participants provided their estimates via VE and TRT. To enhance the participants’ understanding of their estimations, participants were encouraged to use an analogy: envisioning themselves being present during the video shoot.

Unless explicitly stated otherwise, all references to *clip duration* and *original duration* apply to both VE and TRT.

***Additional variables***

Additional exploratory analyses were conducted and included the variables age, gender, use of counting strategies, self-reported memory of previous estimates, ease and confidence of estimations, and decimal precision in estimates. Results will be presented in the SI, Section E.

### Procedure

The study was conducted online via Unipark and had to be completed on a PC or laptop. After providing informed consent in accordance with the Declaration of Helsinki, participants were instructed to conduct the experiment in a quiet environment without clocks or distractions. They were informed that they would view videos at varying speeds and lengths.

To ensure clarity, participants received detailed explanations of the instructions, the different tasks, and estimation methods, followed by practice estimations using four sample videos at different speeds ($\text {x2.0}^{(-)}$, $\text {x3.0}^{(-)}$, $\text {x3.0}^{(+)}$, $\text {x1.0}$).

Participants were reminded to pay close attention to the specific type of estimation queried. They were asked to estimate each video independently, without referring to prior answers, and were assured that the task aimed to assess subjective perception rather than precision, discouraging strategies like counting. Participants viewed 15 videos in a randomized order, each preceded by a fixation cross. After each video, they provided one of the previously introduced five estimations, without knowing in advance which estimate would be required (i.e., *video speed*, *original duration (VE or TRT)*, or *clip duration (VE or TRT)*, see Fig. 1).

Participants did not receive feedback on their estimates to avoid influencing their future responses. The study concluded with a brief exit questionnaire assessing demographic and the additional variables, estimation strategies and potential technical issues. The entire procedure lasted an average of 23 minutes.

### Data preprocessing

Analysis and visualization were performed using MATLAB (Mathworks, 2023), SPSS (IBM, 2023) and CorelDRAW (Corel Corporation, 2023). Data and analysis scripts are provided on OSF (https://osf.io/9asjz/).

***Data exclusions and outliers***

Data were filtered following predefined exclusion criteria detecting incorrect usage of the TRT and video speed estimation task and an outlier analysis following Sperl et al. (2021). Detailed information about these preprocessing steps is provided in the SI, Section A.

***Variable calculations***

Mean ratings were computed for each video speed condition across all three video clips. To account for varying video durations and maintain a consistent error rate (Brown, 1995), percentage deviations from the true value (estimated value/true value * 100) were used as in Hammerschmidt et al. (2021) instead of difference scores as seen in previous studies (Sperl et al., 2021; Hüttner et al., 2023). This method ensures that deviations in duration judgments are equally weighted, regardless of video length. Values above 100% indicate overestimation, while values below 100% indicate underestimation. While deviations focus on error magnitude and direction, accuracy measures (reported in SI, Section B) provide insights into the overall correctness of judgments, offering a broader understanding of duration estimations.

For the *video speed* variable, a transformation of estimates was necessary. Since both slow-motion and time-lapse speeds were reported as positive factors (greater than 1), a linearization of this V-shaped function was applied to facilitate comparisons and statistical analysis.

Because interpreting relative distances on this linearized scale presents challenges^5^, a percentage deviation measure was not used for *video speed*. Instead, a deviation score (estimated speed - true speed) was calculated, where positive values indicate an overestimation and negative values indicate an underestimation of video speed.

### Data analysis

Significance levels were determined through two-sided testing at the $\upalpha$ = .05 level in all analyses. Confidence intervals (CIs) and effect sizes, including partial eta squared ($\upeta _{\text {p}}^{2}$), Pearson’s *r*, and Cohen’s *d*, are reported.

The Shapiro-Wilk test revealed non-normal distributions for most deviation variables. However, parametric tests were justified by the large sample size, as the central limit theorem mitigates this issue (e.g., Blanca, Alarcón, Arnau, Bono, & Bendayan, 2017; Bortz & Schuster, 2010; Havlicek & Peterson, 1976; Pagano, 2010; Schmider, Ziegler, Danay, Beyer, & Bühner, 2010; Wilcox, 2013). These tests enabled more precise analysis, such as evaluating differences in seconds, which rank-based methods cannot achieve.^6^

***Repeated measures analysis of variance***

The main analysis involved a one-way rmANOVA for each dependent variable: *video speed*, *original duration (VE)*, *original duration (TRT)*, *clip duration (VE)*, and *clip duration (TRT)* with video speed condition (slow motion, original speed, and time lapse) as the independent variable. Greenhouse-Geisser corrections were applied where Mauchly’s test indicated sphericity violations, and the Bonferroni method was used to correct for multiple comparisons. One-sample *t* tests were conducted to assess whether the means of estimations significantly deviated from the true values by testing the differences against zero.

***Correlations***

A pairwise Pearson product moment correlation analysis was conducted to gain a deeper understanding of the correlations among the five variables, using all valid cases.

***Estimation methods***

To compare differences between estimation methods (VE vs. TRT), paired-sample *t* tests and Pearson correlation analyses were performed.

## Results

### Deviations across video speed conditions (H1)

**Fig. 2:**
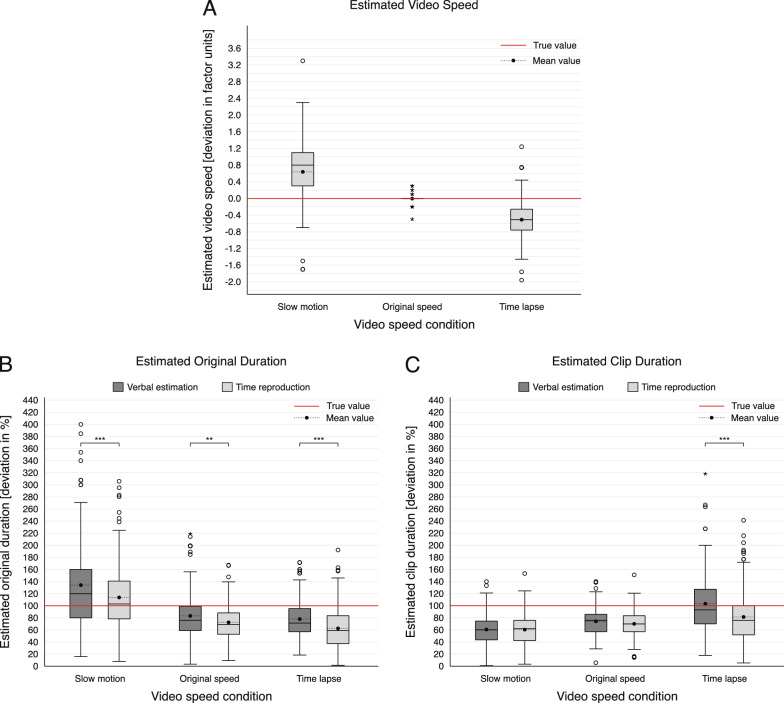
Distribution of deviations in duration and speed estimations across video speed conditions. Panel A displays the deviations in video speed estimations, Panel B in original duration estimations, and Panel C in clip duration estimations, separated by estimation method. The box represents the interquartile range, with the median as a line and the mean shown as a black dot. Outliers (values exceeding three interquartile ranges from the 75th or 25th percentile) are marked as individual points, with extreme values indicated by stars. The true value for each condition is marked by a red line. The sample size varies by condition and is detailed in Table 3. Significance brackets in Panels B and C denote statistically significant differences between VE and TRT. See also Sect. Measurement Methods (Verbal Estimation vs. Reproduction Task) for detailed analyses. ^**^p < .01. ^***^p < .001.

Figure 2 visually presents the deviations for the five main variables across the video speed conditions using box plots.

The results of the rmANOVAs for the five main dependent variables are presented below in the running text, while detailed results from Bonferroni-corrected post hoc *t* tests are presented in Table 2. A comparison between deviations and accuracy across video speeds for each dependent variable is available in the SI, Fig. S2.

***Video speed***

Significant differences in mean deviations were found across video speed conditions, *F*(1.29, 143.48) = 131.23, *p* < .001, $\upeta _{\text {p}}^{2}$ = .54, representing a large effect size. Post hoc comparisons showed that participants significantly overestimated video speed in slow motion (*M* = 0.64 factor units, *SD* = 0.66), slightly underestimated it at original speed (*M* = -0.01 factor units, *SD* = 0.09), and underestimated it in time lapse (*M* = -0.50 factor units, *SD* = 0.53). Differences between all conditions were significant, all *ps* < .001 (Fig. 2A).

***Original duration***

***Verbal Estimation*** Significant differences in original duration deviations across video speed conditions were found, *F*(1.55, 266.15) = 88.08, *p* < .001, $\upeta _{\text {p}}^{2}$ = .34, representing a large effect size. The slow-motion condition showed the largest deviations (*M* = 132.68%, *SD* = 71.57), followed by original speed (*M* = 81.91%, *SD* = 36.52), and time lapse (*M* = 77.51%, *SD* = 34.00), all *p*s < .001. No significant difference was found between original speed and time lapse, *p* = .486 (Fig. 2B).

***Time Reproduction*** The deviations for original duration (TRT) also varied significantly, *F*(1.56, 259.40) = 89.66, *p* < .001, $\upeta _{\text {p}}^{2}$ = .35, representing a large effect size. The highest deviations occurred in slow motion (*M* = 113.80%, *SD* = 56.21), followed by original speed (*M* = 73.00%, *SD* = 26.55) and time lapse (*M* = 64.00%, *SD* = 33.16), all *p*s $\le$ .006 (Fig. 2B).

***Clip duration***

***Verbal Estimation*** Significant differences in clip duration deviations were found, *F*(1.33, 226.47) = 102.39, *p* < .001, $\upeta _{\text {p}}^{2}$ = .38, representing a large effect size. Time lapse had the highest deviations (*M* = 102.45%, *SD* = 45.81), followed by original speed (*M* = 74.17%, *SD* = 23.94) and slow motion (*M* = 60.30%, *SD* = 22.63), all *ps* < .001 (Fig. 2C).

***Time Reproduction*** Clip duration deviations in TRT also showed significant differences, *F*(1.64, 264.65) = 33.36, *p* < .001, $\upeta _{\text {p}}^{2}$ = .17, representing a large effect size. The lowest deviations occurred in slow motion (*M* = 61.12%, *SD* = 23.68), followed by original speed (*M* = 69.88%, *SD* = 21.00), with the highest in time lapse (*M* = 84.31%, *SD* = 40.04), all *ps* < .001 (Fig. 2C).

**Table 2:** Post hoc comparisons for video speed effects on speed and duration estimations for the deviations

Variable	Comparison	$M_{\text {Diff}}$	*SE*	*p*	*n*	95% CI	*d*
Speed factor	SM vs. OS	0.65	0.06	< .001	112	[0.50, 0.80]	0.99
	SM vs. TL	1.14	0.93	< .001	112	[0.91, 1.36]	1.16
	OS vs. TL	0.49	0.05	< .001	112	[0.37, 0.61]	0.92
Original duration (VE)	SM vs. OS	50.78	5.15	< .001	173	[38.32, 63.24]	0.75
	SM vs. TL	55.18	5.26	< .001	173	[42.46, 67.89]	0.80
	OS vs. TL	4.40	3.13	.486	173	[−3.18, 11.98]	0.11
Original duration (TRT)	SM vs. OS	40.81	4.01	< .001	167	[31.12, 50.50]	0.79
	SM vs. TL	49.85	4.77	< .001	167	[38.31, 61.39]	0.81
	OS vs. TL	9.04	2.89	.006	167	[2.05, 16.04]	0.24
Clip duration (VE)	SM vs. OS	−13.88	1.64	< .001	171	[−17.83, −9.92]	−0.65
	SM vs. TL	−42.15	3.58	< .001	171	[−50.80, −33.50]	−0.90
	OS vs. TL	−28.28	3.40	< .001	171	[−36.50, −20.05]	−0.64
Clip duration (TRT)	SM vs. OS	−8.76	2.16	< .001	162	[−13.98, −3.53]	−0.32
	SM vs. TL	−23.19	3.35	< .001	162	[−31.29, −15.08]	−0.54
	OS vs. TL	−14.43	2.96	< .001	162	[−21.59, −7.27]	−0.38

*Note.* Bonferroni-corrected post hoc tests for multiple comparisons are reported following the significant rmANOVA. SM = slow motion; OS = original speed; TL = time lapse; VE = verbal estimation; TRT = time reproduction task; CI = confidence interval

***Deviations compared to true values***

Detailed statistical outcomes from one-sample *t* tests, evaluating deviations from true values, are presented in Table 3.

**Table 3:** Deviation analysis: descriptive statistics and one-sample *t* test results for duration and speed estimations

Variable	Deviations		$\Delta$		*t*	*df*	*p*	*d*
	*M*	*SD*	[factor units]					
Video speed								
Slow motion	0.64	0.72	+0.64		10.71	145	< .001	0.89
Original speed	–0.00	0.09	–0.00		–0.57	139	.573	–0.00
Time lapse	–0.51	0.51	–0.51		–12.49	155	< .001	–1.00

Variable	Deviations		$\Delta$		*t*	*df*	*p*	*d*
	*M*	*SD*	[%]	[s]				
Original duration (VE)								
Slow motion	134.20	72.64	+34.20	+2.05	6.23	174	< .001	0.47
Original speed	83.26	38.43	−17.74	−1.07	−5.76	174	< .001	−0.44
Time lapse	77.88	34.26	−22.12	−1.37	−8.52	173	< .001	−0.65
Original duration (TRT)								
Slow motion	113.74	56.05	+13.74	+0.82	3.18	167	.002	0.25
Original speed	72.46	27.47	−28.54	−1.69	−13.18	172	< .001	−1.00
Time lapse	62.54	34.36	−37.46	−2.31	−14.50	176	< .001	−1.09
Clip duration (VE)								
Slow motion	60.63	23.23	−39.37	−5.49	−22.55	176	< .001	−1.70
Original speed	74.22	23.92	−25.78	−1.61	−14.18	172	< .001	−1.08
Time lapse	103.30	46.03	+3.30	+0.08	0.95	173	.346	0.07
Clip duration (TRT)								
Slow motion	60.46	23.96	−39.54	−5.54	−21.45	168	< .001	−1.65
Original speed	69.97	21.59	−30.03	−1.87	−17.92	165	< .001	−1.39
Time lapse	81.39	41.50	−18.61	−0.51	−5.92	173	< .001	−0.45

*Note.* The table presents the mean values for the deviations of each dependent variable across different video speed conditions, detailing both the percentage deviations and deviations in seconds relative to the true value. Overestimations are denoted with ’+’, while underestimations are marked with ’-’. Additionally, the table includes results from *t* tests that compare the deviations with the true value (i.e., 100% for duration deviations and 0 for speed deviations, respectively), i.e., testing the differences against zero. Note that the means and standard deviations differ from the rmANOVA results due to varying degrees of freedom. In rmANOVA, all values for all three speed conditions must be present, whereas in the one-sample *t* tests, individual data points are analyzed independently.

### Relations between duration estimations and video speed estimates (H2)

Positive correlations were found between *original duration* and *clip duration* estimations (for VE and TRT) across all speed conditions (all *ps* < .001). Notably, duration estimations and *video speed* estimations were uncorrelated, except for a small positive correlation between *original duration (VE)* and *video speed* estimation in slow motion (*r* = .21, *p* = .010). This suggests that higher estimations of the *original duration (VE)* in slow motion were associated with a higher perceived *video speed*, indicating a lower estimation of the slow-motion factor. For a concise summary of the correlations within the different video speeds, refer to the SI, Tables S5, S6, S7.

### Measurement methods (verbal estimation vs. reproduction task)

***Relationship between measurement methods***

Moderate correlations were observed between VE and TRT for *original duration* in both slow motion (*r*(165) = .42, *p* < .001) and time lapse (*r*(172) = .33, *p* < .001), while a smaller correlation was found in original speed (*r*(169) = .18, *p* = .019). These results suggest that participants who provided higher VE estimates tended to report higher TRT estimates as well.

For *clip duration*, small to moderate correlations were found in slow motion (*r*(167) = .23, *p* = .003), time lapse (*r*(169) = .16, *p* = .033) and original speed (*r*(162) = .36, *p* < .001), suggesting a consistent relationship between VE and TRT across all speed conditions. A comparison of VE and TRT estimations using dependent *t* tests is summarized in Table 4.

Significant differences were found in all speed conditions for *original duration*, with TRT generally yielding lower estimates, particularly in slow motion where the effect size was moderate (*d* = 0.30). In time lapse, VE was more accurate, demonstrating a larger deviation from TRT (*d* = 0.41). For *clip duration*, no significant difference was observed in slow motion, while original speed showed only a marginal difference. In time lapse, VE estimates were again closer to the true value than TRT (*d* = 0.39).

These findings indicate that VE generally leads to more accurate estimates in faster conditions (original speed and time lapse), while TRT is more aligned with true values in slow motion.

**Table 4:** Means, standard deviations, and dependent *t* test statistics for deviation variables

Variable	VE		TRT		*t*	*df*	*p*	95% CI	*d*
	*M*	*SD*	*M*	*SD*					
Original duration									
SM	134.83	73.25	113.38	56.02	3.90	166	< .001	[10.59, 32.30]	0.30
OS	83.40	38.79	72.73	27.30	3.23	170	.001	[4.15, 17.21]	0.25
TL	77.88	34.26	61.80	33.62	5.40	173	< .001	[10.20, 21.96]	0.41
Clip duration									
SM	61.55	22.96	60.46	23.96	0.48	168	.630	[–3.35, 5.52]	0.04
OS	74.09	23.41	70.24	21.54	1.94	163	.054	[–0.07, 7.76]	0.15
TL	103.30	46.41	81.26	40.90	5.09	170	< .001	[13.50, 30.60]	0.39

*Note.* Mean deviations of original and clip duration, verbal estimation vs. time reproduction task, across different video speed conditions are presented. SM = slow motion; OS = original speed; TL = time lapse; VE = verbal estimation; TRT = time reproduction task; CI = confidence interval.

### Potential roots of a distorted sense of original duration

Based on the idea that both *clip duration* and *video speed* are needed to make inferences about the *original duration*, we explored the relationship between these three variables in a further step. Mathematically, *original duration* can directly be calculated from the other two, by multiplying the clip duration by its respective video speed. However, since we did not provide participants with information about these parameters, they had to form their own interpretations of these parameters. In this additional analysis, we aimed to investigate whether their distorted sense of original duration originated from their inaccurate perception of video speed and clip durations. Therefore, we used participants' estimated values of clip duration and video speed to calculate the original duration they should have experienced, when basing their inference on their own, albeit incorrect, perceptions. This *calculated original duration* was then compared to the actual estimates provided by the participants and the true original durations.

In time lapse, this calculated original duration closely matched the estimations they actually provided with an accuracy of approximately 150 ms for both VE (*M* = -0.14 s, *SD* = 2.16, *t*(151) = -0.67, *p* = .507) and TRT (*M* = -0.16 s, *SD* = 2.80, *t*(152) = -0.70, *p* = .487). Also in the original-speed condition, the calculated original duration was close to what participants actually provided as estimation via VE (*M* = -0.34 s, *SD* = 1.86, *t*(137) = -2.17, *p* = .032) and TRT (*M* = -0.33 s, *SD* = 1.34, *t*(131) = -2.86, *p* = .005). Note that although statistically significant, the difference remained small, deviating by only about 330 ms from participants’ reported values. For time lapse and original speed, it can thus be suggested that their own, yet erroneous, perception of clip duration and video speed indeed led participants to their deviation in original duration perception.

A different pattern emerged in the slow-motion condition, where, interestingly, the calculated duration rather aligned with the true original duration (VE: *M* = -0.05 s, *SD* = 4.43, *t*(145) = -0.14, *p* = .890 / TRT: *M* = -0.10 s, *SD* = 3.68, *t*(141) = -0.32, *p* = .751), than with the estimations participants provided (VE: *M* = -2.07 s, *SD* = 4.32, *t*(144) = -5.77, *p* < .001 / TRT: *M* = -1.11 s, *SD* = 4.11, *t*(141) = -3.21, *p* = .002).

**Fig. 3:**
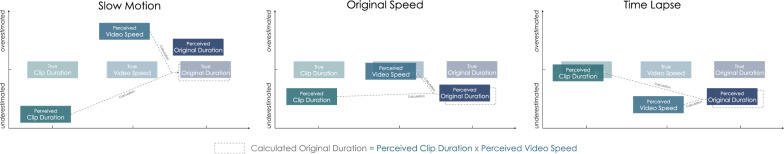
Summary of the results. This figure illustrates the deviation of participants’ perceived durations and speeds (darker boxes in the foreground) from the true values (lighter boxes in the background) in slow motion (left), original speed (middle) and time lapse (right). The closer the boxes are placed to each other or even overlap, the more accurate participants’ estimations were (schematic illustration, VE and TRT is summarized). Dashed lines highlight that perceived clip duration and video speed estimates were mathematically combined to derive a corresponding “calculated” value for original duration (see also dashed box) showing the original duration participants should have perceived when using their own, yet biased, perception of clip duration and video speed (dark boxes)

Even though participants’ own, albeit inaccurate, perceptions of video speed and clip duration should - at least mathematically - still lead them to the correct original duration, they still overestimated the original duration. Thus, while working surprisingly well for time lapse and original speed, at least in the slow-motion condition, mathematical-physical relations alone cannot fully capture the mental processes underlying their judgments. This relationship is illustrated in Fig. 3.

## Discussion

The objective of this study was threefold: first, to systematically explore how individuals perceive video speed, clip duration, and the original duration of video scenes across different speed conditions (slow motion, original speed, time lapse); second, to conduct a comprehensive correlation analysis to understand how these subjective estimates are interrelated; and lastly to assess different time measurement methods (VE vs. TRT) to address discrepant results on time perception in existing research.

Overall, our data indicate that participants’ perception of the three key variables is notably biased with considerable deviations from the true values. The direction of these biases, i.e., the general patterns of under- vs. overestimation, was largely similar across both measurement methods (VE and TRT) and is illustrated in Fig. 3, which summarizes the key results of this study.

While *video speed* was perceived as fairly accurate in the original-speed condition (see overlap of the blue video speed boxes in the middle panel of Fig. 3), video speed was significantly overestimated in slow motion (left panel) and underestimated in time lapse (right panel). Hence, in other words, when a video was altered in its speed, participants perceived the extent of that speed manipulation as less extreme than it actually was.

For *clip duration*, it was visible that the slower the video was played, the shorter its duration was experienced. In both slow motion and original speed, participants underestimated clip duration, whereas in time lapse, it was perceived as longest and fairly accurate (see overlap of the greenish clip duration boxes).

Regarding *original duration*, this pattern was reversed. It was overestimated in slow motion (aligning with the overestimation reported in Sperl et al. (2021); see dark blue box in left panel) while original durations were perceived as shorter than they actually were in the original-speed (middle panel) and in the time-lapse condition (right panel).

While estimates of clip duration and video speed might be derived directly from monitoring the passing time or observing the motion speed of the stimulus content, estimating the *original duration* might be a more complex process which requires several steps. Instead, participants might first estimate the other two variables (clip duration and video speed) to then combine them to gain a percept of the time that originally passed. If true, one might expect that participants’ sense of original duration can be (roughly) derived from multiplying their perceived clip duration by perceived video speed. Indeed, this calculation results in values that closely match the original duration estimations participants actually provided (visible in the overlap of the dashed boxes with the dark blue perceived original duration boxes in middle and right panel), suggesting that their distorted sense for original duration might indeed be rooted in their own, yet erroneous, perceptions of clip duration and video speed. This is the case for the original-speed and time-lapse condition. However, in the slow-motion condition, this calculation leads to values close to the true original duration instead, while participants still clearly overestimated original duration (possibly reflecting an overcompensation mechanism). This indicates that time perception in slow motion, specifically regarding the original duration, clearly differs from other video speeds and cannot be explained by mathematical relations alone.

### Video speed, original duration and clip duration perception across different speed conditions

#### Estimation of video speed

Our results of biased *video speed* estimations highlight the difficulties humans have with perceiving altered video speeds (de’Sperati & Baud Bovy, 2017; Rossi et al., 2018; Sperl et al., 2021; Hüttner et al., 2023). On average, participants’ estimations were less extreme than the actual speed changes, displaying a tendency toward the original speed: in time lapse, participants consistently underestimated *video speed*, making the videos appear slower than their true speed, while in slow motion, *video speed* was overestimated ('faster'), supporting the overestimation bias for *original duration* observed by Sperl et al. (2021).

Although participants were fairly accurate in estimating *video speed* in the original-speed condition, indeed representing a rather simple task, previous studies using speed adjustment tasks, such as Zuliani et al. (2019) and Rossi et al. (2018), found underestimations already at original speed. This suggests that different methods, such as speed adjustments versus verbal estimations, can yield different results.

Interestingly, *video speed* estimations exhibited a pattern similar to that of Vierordt’s Law (Vierordt, 1868), a well-known principle in time perception research. This law posits that longer durations tend to be underestimated, while shorter durations are overestimated, with an indifference point in between. Vierordt’s Law, when interpreted as the Central Tendency Effect (Hollingworth, 1909), is thought to arise from perceptual judgments regressing toward the midpoint of a stimulus range (Lejeune & Wearden, 2009). In the present study, the original speed likely served as such a midpoint, biasing the speed judgments. This concept aligns with Mather et al. (2017), suggesting adaptation to an internal standard as a reference point. Watching a slow-motion or time-lapse video for 30 seconds led to a perceptual shift, causing a subsequent clip to be perceived as too fast or too slow, respectively. Similar effects are also observed in time perception studies (e.g., Bausenhart, Dyjas, & Ulrich, 2014; Grivel et al., 2011; Jazayeri & Shadlen, 2010; Roy, Burns, & Radzevick, 2019), suggesting that time perception mechanisms can extend to video speed perception as well.

#### Estimation of duration (original duration and clip duration)

The present study also found that estimations of *original duration* and *clip duration* differed across speed conditions. The only exception here was the estimation of *original duration (VE)* between original speed and time lapse, where no significant difference was found.

Except for the nearly accurate *clip duration (VE)* in time lapse, clip durations were generally underestimated, replicating Rossi et al.'s (2018) findings with TRT, and additionally confirmed here for VEs, suggesting distorted temporal perception. The *original duration* estimations in slow motion were significantly overestimated with both measurement methods (VE and TRT), extending the overestimation bias documented by Sperl et al. (2021) and Schütz et al. (2021) for VEs to TRT as well.

Notably, the video speed of the presented video should actually be completely irrelevant for estimating clip duration. Nonetheless, (altered) video speeds still seem to distort our perception of clip duration. While *clip duration* was generally underestimated, but increased with increased video speed, estimations of *original duration* showed an inverse pattern. Original duration estimations decreased as video speed increased and were most accurate for videos presented at original speed (see Fig. 2 and Fig. 3).

Overall, the nearly consistent significant deviations from the true value confirm findings from traditional time research that time perception is subjective, variable, and prone to distortions (Pariyadath & Eagleman, 2007; Ebert & Prelec, 2007; Michon & Jackson, 1985), and this is also evident in the context of time perception when video speed is altered.

In this study, participants only received task instructions (duration or speed estimation) after viewing, which minimizes the influence of task-induced cognitive processes such as attention allocation, cognitive load, or information processing requirements (Tse et al., 2004; Cicchini & Morrone, 2009; Zang et al., 2022; Block et al., 2010; Zakay & Block, 1997; Thomas & Weaver, 1975). Thus, differences in perceived outcomes could be rooted in post-encoding cognitive processes, such as retrieval and interpretation of stored information, rather than purely perceptual mechanisms.

***Broader perspective: stimulus speed vs. video speed***

While video speed is technically irrelevant for estimating the duration of the viewed clip, it appears to subconsciously affect duration estimations. The observed increase in *clip duration* estimates from slow motion to time lapse can be explained by established time perception models. Traditional time perception research shows that faster-moving stimuli are perceived as lasting longer than slower-moving stimuli (Brown, 1995; Goldstone & Lhamon, 1974; Kanai et al., 2006; Kaneko & Murakami, 2009; Kline & Reed, 2013; Roelofs & Zeeman, 1951).

Internal clock models (Rammsayer & Ulrich, 2001; Treisman, 1963; Church, 2003) suggest that perceived duration depends on the accumulation of pulses within a given interval: more pulses indicate a longer perceived duration, while fewer pulses suggest a shorter one. Our findings align with this model, as faster speeds in time lapse led to overestimated *clip durations*, while slower speeds in slow motion led to underestimation. Event change models (Fraisse, 1963; Block, 1978) emphasize segmentation of events. More frequent segmentation due to higher speeds, causing more rapid changes, result in longer perceived durations (Brown, 1995). This pattern is evident in the present study, particularly in the time-lapse condition, where faster speeds could lead to increased event segmentation and longer perceived *clip durations* compared to the original-speed and slow-motion conditions.

However, the distinction between stimulus speed and video speed is still important. Stimulus speed typically refers to the velocity of simple, artificial visual stimuli. In contrast, video speed involves a broader range of factors, including meaningful interpretation, real-world cues or familiarity with altered video content that allow us to determine a deviation from the real-life speed (which exists for videos, but not for stimuli such as geometric shapes).

In a study briefly outlined in a conference abstract which explored time distortions in slow-motion videos, Eagleman (2004) mentions a possible explanation for the effect of video speed on duration estimates. He showed that the perceived duration of flashes in a slow-motion sequence with natural biological movements was on average 27% shorter than in the same sequence at original speed. It is suggested that the brain automatically makes compensatory adjustments when watching a video with altered speed by speeding up the perception of video speed in slow motion and slowing it down in time lapse. This leads to results that exactly match those of our study: clip duration was underestimated in slow motion, while it was overestimated in time lapse.

### Correlational relationships between video speed, original duration and clip duration estimations

We observed significant positive correlations between *original duration* and *clip duration* estimations across all video speeds and measurement methods with higher *original duration* estimations being associated with higher *clip duration* estimates, indicating common underlying cognitive processes.

Surprisingly, duration estimations, irrespective of the measurement method or speed condition, did not show any significant correlations with corresponding *video speed* estimations. Intriguingly, there was only one exception: in the slow-motion condition, a significant positive correlation between *original duration estimation (VE)* and *video speed* estimations was found. Here, a greater overestimation of the *original duration (VE)* was associated with a larger overestimation of *video speed* (i.e., an underestimation of the slow-motion factor). This particular finding again aligns with the assumption of Sperl et al. (2021), who focused on original duration estimations of slow-motion videos using VE. They suggested that the overestimation bias in slow motion is more likely due to an overestimation of video speed rather than the original duration itself.

The lack of correlation between *video speed* and duration estimates across other conditions echoes the findings of Rossi et al. (2018), who reported no relationship between video speed perception and clip duration estimates (original duration was not assessed in their study). This supports their notion that distinct cognitive systems may govern the perception of video speed and duration. This is interesting, because at least estimating *original duration* should require a sense of altered speed in order to understand how long an action would have lasted in real time.

### Comparison of measurement methods: verbal estimation vs. time reproduction task

Significant positive correlations between the measurement methods were observed for both the estimation of *original duration* and *clip duration* across all speeds. This indicates that with higher VE, TRT estimations also increased (see Asaoka & Watanabe, 2015; Du Preez, 1963). At the same time, we observed significant differences between the time estimation methods, which was fully the case for *original duration* estimates across all speeds, but only partially for *clip duration* estimates.

Remarkably, all estimations made with TRT were shorter, consistent with findings from prior studies (Damsma et al., 2021; Asaoka & Watanabe, 2015; Clausen, 1950; Brown, 1985; Hornstein & Rotter, 1969; McConchie & Rutschmann, 1971; Riemer et al., 2012). Across all video speed conditions, VE estimations tended to be closer to the true values, except in slow-motion conditions.

The systematic underestimation of duration in TRT is widely reported (Droit-Volet, 2010), with Riemer et al. (2012) attributing this to cautious decision-making under uncertainty and Zakay and Block (1997) to attentional resoures. Participants tend to end the task early in uncertain situations, aiming to avoid overshooting the target interval.

While separating perceptual from motor influences in TRT is challenging (Droit-Volet, 2010), VE is prone to individual biases, such as a preference for estimates ending in “0” or “5” (see also SI, Section E) (Spencer, 1921; Yerkes & Urban, 1906; Asaoka & Watanabe, 2015). This bias may explain why VE results aligned more closely with true values, as most of the video clip durations used in this study ended in these digits.

Given the consistent correlation between VE and TRT across all video speeds and the uniformity of stimulus presentation, the observed differences in estimation magnitude may reflect task-implicit factors such as decision-making processes or response patterns rather than differences in time perception itself (Riemer et al., 2012; Droit-Volet, 2010).

### Methodological considerations

The stimulus videos, while serving as proxies for real-life activities, were not specifically designed to assess the effects of clip content on speed and time perception. However, recognizing that content can influence such ratings (Rossi et al., 2018; Sperl et al., 2021), the chosen clips were carefully created and selected to be comparable (see also SI, Section D). It must be considered that even if we aimed to provide videos that allowed participants to draw on their experience and physical laws, there is still no universally applicable benchmark for the temporal standard of executing an everyday action from which changes in speed can be derived (Lacquaniti et al., 2015).

The factors by which the videos were slowed down or sped up, along with the video lengths, were calculated and chosen to ensure suitable length for both slow-motion and time-lapse videos. Given that the video lengths were inherently determined by the speed factors when desiring to hold the clip constant across different speed levels, with time-lapse videos associated with shorter duration and longer slow-motion videos, it exceeds the scope of this study to also investigate potential effects of different clip lengths between conditions. However, findings from research on time perception hint at an interaction between stimulus speed and experienced duration (Brown, 1995; Tayama et al., 1987; Fraisse, 1962).

In addition, stimulus length is known to influence the extent to which durations are under-/overestimated (e.g., Riemer & Wolbers, 2020; Vierordt, 1868). While Caruso et al. (2016) ruled out longer exposure as the sole cause for increased duration and intentionality effects when watching slowed-down videos, prolonged exposure in this condition may still influence participant ratings. For a broader generalization of the findings, employing a range of video speeds and lengths, and examining and comparing their effects, along with using videos of equal length across different speed conditions, would be beneficial. However, such a design would inevitably result in variations in clip content at different implemented video speeds and therefore also provide cues about the video speed due to the fact that actions do not always reach completion. Additionally, repeated viewings necessary for collecting different variables could invalidate clip-length estimates (since participants would likely realize after a few trials that the clip-length rating would be always the same (e.g., 5 s) irrespective of speed condition).

To address the potential confound of stimulus duration, Sperl and Liepelt (2024) recently conducted a study where video duration was held constant across different video speed conditions by using monotonous repetitive actions in their videos. Their findings demonstrate that the over-/underestimations of video speed and clip duration occurred independently of stimulus duration and, in line with the present study, that even an irrelevant video speed characteristic can influence participants’ temporal experience of how long a video is.

### Future research and open questions

As this is one of the first systematic investigations into key variables influencing human video speed perception, replication studies, using also different stimuli and video speeds, are encouraged to strengthen foundation for future research.

Further exploration of purely retrospective versus prospective paradigms could offer deeper insights into the underlying processes as prospective estimates rely on attentional encoding, whereas retrospective estimates are shaped by memory retrieval (Block et al., 2010; Block & Zakay, 1997, 2001; Zakay & Block, 2004).

Moreover, investigating the impact of serial effects, such as how video speed in one trial might affect subsequent judgments, could offer valuable insights. Research suggests that biases in the first estimation (Roy et al., 2019) or previously seen video speeds (Mather et al., 2017; Grivel et al., 2011) can influence later perceptions and tasks. While this was not the focus in the present study, and a strategic advanced randomization was employed to counteract serial effects, this remains a promising area for future research.

In addition, exploring the role of clip content and in this context also familiarity (both visual and motor) with the displayed actions merits attention (see also Rossi et al., 2018).

The rather incorrect estimation of clip duration raises the question of whether providing information on clip duration - similar to providing video speed information (Sperl et al., 2021) - could reduce the overestimation bias of original duration in slow motion. Additionally, the influence of post-perceptual processes that may model clip and original duration, leading to the observed inverse pattern, and the differences between measurement methods, should be further explored.

### Practical implications

The findings of this study reveal significant misperceptions in time and video speed perception when video speed is altered and emphasize the need for cautious use and application of video technologies in the current digital landscape. In critical contexts such as sports officiating and legal proceedings, slow-motion footage is known to (even subconsciously) distort our temporal perception and increase perceived intentionality, potentially leading to biased judgments. Specifically, viewers evaluating video evidence are observed to perceive actions as more intentional and willful and to apply stricter sanctions when inspecting footage in slow motion compared to original speed (Caruso et al., 2016; Spitz et al., 2018). Being aware of how perceptual distortions occur can help to reduce or even prevent such biases. Future research might also engage with developing training programs for professionals like referees and legal personnel that help to prevent misapplications.

For media professionals - whether in advertising, journalism, or education - training on the ethical use of video speed alterations is also crucial. While these techniques can drive engagement, such as fostering urgency in climate action campaigns through time lapse (Sheng et al., 2022), they may also manipulate viewer perception and influence consumer behavior (Yin et al., 2021). Promoting awareness and ethical usage can prevent misuse while maximizing the positive potential of video speed manipulations.

However, at the same time it is important to acknowledge, that our knowledge of how humans perceive altered video speeds is still at an early stage. To advance this field, it is crucial to also conduct fundamental research to gain an understanding of the cognitive processes underlying the reported biases.

### Conclusion

The present study systematically examined human perception of altered video speeds and the estimation of original and clip durations across varying speed conditions. The findings revealed biases in speed perception consistent with prior research (e.g., de’Sperati & Baud Bovy, 2017; Sperl et al., 2021). Participants underestimated video speed in time-lapse conditions and overestimated it in slow motion. Participants’ estimates seemed to tend toward an internal perceptual standard (Mather et al., 2017), illustrating the Central Tendency Effect, which possibly reflects a compensatory adjustment already during watching these temporally distorted videos.

A similar pattern was observed in clip duration estimates, while an inverse pattern appeared for original duration, showing an overestimation bias in slow motion (Sperl et al., 2021) and underestimation in time lapse. From a mathematical point of view, participants’ duration and speed estimates align logically, suggesting perceived original duration may arise from integrating (inaccurately) perceived clip duration and video speed. However, while this relationship holds true for original and time-lapse videos, it breaks down for slow-motion videos. This discrepancy might point to distinct cognitive mechanisms governing slow-motion judgments, potentially involving overcompensation or altered perceptual integration.

The positive correlation between clip and original duration estimations suggests a shared cognitive mechanism, though video speed perception did generally not correlate with either duration, except for one association in the slow-motion condition (which might again highlight the singularity of slow-motion perception).

Overall, this study displays the complexity of the interplay between perceptual and cognitive factors in time and video speed perception, varying based on the specific duration being estimated (clip vs. original) and the method of measurement used.

## Supplementary Information

Supplementary file 1.

## Data Availability

The datasets generated and analyzed during the current study, materials and codes are available in the Open Science Framework repository, https://osf.io/9asjz/.

## Footnotes

1 Note that an a-priori power analysis was conducted with G*Power 3.1.9.7 to determine the minimum targeted sample size, which was calculated as *n* = 138 (based on medium effect size, power of .95, i.e., two-sided testing, tested for all main analyses, i.e., rmANOVAs, correlations and *t* tests).

2 Note that the factor of 2.3 for acceleration and deceleration was chosen to ensure time-lapse videos were not too short (allowing action recognition) and slow-motion videos were not too long (preventing participant fatigue). Symmetric speed variations were selected for comparability, though participants were unaware of this.

3 The decision to use a factor instead of a percentage value for *video speed* estimates was due to the limited range of percentages. In the slow-motion direction only values between 0 and 100% are possible, whereas in the time-lapse direction values may exceed 100%. Consequently, a 15% deviation in time-lapse direction, for instance, has less impact than a 15% deviation in the slow-motion direction, owing to differing weighting mechanisms. This inconsistency underscores the more precise representation of speed changes with video speed factors, as they are symmetrical in both slow-motion and time-lapse directions.

4 Previously described as *absolute time* in prior research by Sperl et al. (2021) and Hüttner et al. (2023).

5 Some combinations of estimated and true values result in contradictory or uncalculable results, particularly in cases requiring division by zero. However, since the true video speeds were symmetrically distributed (factor 2.3 for both slow motion and time lapse), the use of percentage measures - unlike for durations - is unnecessary here.

6 Additionally, following the suggestion of an anonymous reviewer and to correct for potential biases in the deviation measure, where underestimations are limited to a range of 0% to 100% while overestimations remain unbounded, a log transformation to the ratio of estimated to true values was performed for the main variables. This adjustment centers the values around 0, providing a more balanced reflection of estimation differences. The analyses with log-transformed values showed no differences in the significance patterns of the rmANOVA, only minor differences were found in the correlations and the comparisons of the measurement methods. Full details of this analysis are available in the SI, Section C.

## Declarations

### Open practices statement

All data and materials including a pre-registration of this experiment are available at https://osf.io/9asjz/.
