# Supplementary material for: Time and video speed perception: a comprehensive investigation of the relation between estimated video speed, clip duration and original duration
Source: Cogn Res Princ Implic. 2025 Jul 1;10:36. doi: 10.1186/s41235-025-00637-2 (PMC12214220; doi:10.1186/s41235-025-00637-2)
Supplement: Supplementary file 1 [file 41235_2025_637_MOESM1_ESM.pdf]

Time and Video Speed Perception: A Comprehensive Investigation of the Relation Between Estimated Video Speed, Clip Duration and Original Duration

Verena Steinhof[1], Anna Schroeger[2,3], Roman Liepelt[1], Laura Sperl[1*]

[1*]Department of General Psychology: Judgment, Decision Making, Action, Faculty of Psychology, University of Hagen (FernUniversität in Hagen), Universitätsstraße 37, 58084 Hagen, North Rhine-Westphalia, Germany.
[2]Department of Experimental Psychology, Faculty of Psychology, University of Gießen (Justus-Liebig-Universität Gießen), Alter Steinbacher Weg 38, 35394 Gießen, Hesse, Germany.
[3]Center for Mind, Brain and Behavior, University of Marburg, Justus Liebig University Giessen and University Darmstadt, Hans-Meerwein-Straße 6, 35032, Marburg, Hesse, Germany.

*Corresponding author(s). E-mail(s): laura.sperl@fernuni-hagen.de; Contributing authors: verena.steinhof@studium.fernuni-hagen.de; anna.schroeger@psychol.uni-giessen.de; roman.liepelt@fernuni-hagen.de;

Supplementary Information

A Additional Methodological Information

A.1 Stimulus Randomization

Table S1 Allocation of Video Speeds for Clips Across Experimental Groups

	Video clip A (dice)	Video clip B (teapot)	Video clip C (match)
Group 1	Slow motion	Original speed	Time lapse
Group 2	Original speed	Slow motion	Time lapse
Group 3	Time lapse	Original speed	Slow motion
Group 4	Slow motion	Time lapse	Original speed
Group 5	Original speed	Time lapse	Slow motion
Group 6	Time lapse	Slow motion	Original speed

Note. The randomization ensures that each participant is exposed to all speed conditions, covers all combinations for a complete dataset, and controls for order effects.

A.2 Data Exclusion and Outliers

Exclusion Prior to Analyses

To control for incorrect executions of the TRT (e.g., values close to zero due to slipping off the key, user error, lack of seriousness), specific data exclusion criteria were defined for the TRT estimates: For the estimates of *original duration (TRT)* and *clip duration (TRT)*, all measurements with a reproduction duration of < 500 ms in the slow motion or original speed condition were excluded. This applied to 12 (7%) and 16 (9%) estimations, respectively. In the case of shorter video clips in the time lapse conditions, a minimum limit of 100 ms was established, which led to the exclusion of one estimation. TRT lower limits were derived from previous studies (500 ms according to Maaß, Riemer, Wolbers, and van Rijn (2019), 100 ms as per Matthews (2011)).

Additionally, all estimations from participants who provided an invalid factor for *video speed* estimation, i.e., a value ≤ 1 for slow motion (27 estimations, equivalent to 15%) or for time lapse (12 estimations, equivalent to 7%), or a value $\neq 1$ for OS (12 estimations, equivalent to 7%), were also excluded from the respective analyses involving these variables. Participants with more than one incorrect *video speed* estimation (i.e. two or three incorrect responses), indicating a lack of understanding of the task, were completely excluded from the analyses involving the *video speed* variable (13 responses, corresponds to 7% of the individuals). These criteria were applied based on the raw values and were pre-determined for exclusion before any analyses were conducted. Practice trials did not enter data analysis either.

Handling Outliers in Calculated Variables

Outlier analysis was conducted using deviation variables (for details on the calculation of deviation variables, see Section 2.6 in the main paper). instead of raw values, to accommodate varying video lengths and account for different video speed categories in the *video speed* variable calculation. The outlier analysis was performed using interquartile ranges, following the methodology outlined in Sperl, Hüttner, and Schroeger (2021) and Hüttner, Sperl, and Schroeger (2023). In the case of duration estimations, including *original duration (VE, TRT)* and *clip duration (VE, TRT)*, 18 estimates (10%, out of 13 participants) that deviated more than three interquartile ranges above the 75th percentile or below the 25th percentile were identified and excluded from analyses involving these variables. Likewise, for the *video speed* variable 30 estimates (17%, out of 28 participants) exceeding three interquartile ranges above the 75th percentile or below the 25th percentile based on the difference values were excluded.

In the exploratory analysis regarding the temporal scaling formulas, the same outliers that were identified on the basis of the deviations were excluded from the raw estimates of the participants that were needed to form the quotient, ensuring consistency across all analyses conducted in this study.

The final dataset, after all exclusions, consisted of 177 participants, who were randomly distributed across six groups (see SI, Table S1) as follows: Group 1 (n = 31), Group 2 (n = 31), Group 3 (n = 27), Group 4 (n = 33), Group 5 (n = 26), and Group 6 (n = 29).

B Additional Analyses for Accuracy as Dependent Variable

Absolute *accuracy* values for the duration estimations were computed based on the deviations. This was done by subtracting 100 from the percentage deviations and taking the absolute value, representing an overall level of accuracy in judgments. That is, a higher value of accuracy represents a larger percentage deviation from the true value compared to smaller values and will be referred to as *accuracy* in the following. For *video speed accuracy*, the absolute value of the difference score was calculated. This measure was employed to assess the accuracy of the estimates, preventing cancellations between overestimations and underestimations. Together, both deviation measures and accuracy measures offer valuable insights into the data, each with its own focus.

B.1 Duration and Speed Estimations of Accuracy

Table S2 Means, Standard Deviations, and One-Way Repeated Measure ANOVA Statistics for Accuracy Variables

Variable	Slow motion		Original speed		Time lapse		*F*	*p*	η_p^2
	M	*SD*	*M*	*SD*	*M*	*SD*			
Speed factor	0.83	0.39	0.03	0.09	0.62	0.38	235.72^{***}	< .001	0.68
Original d. (VE)	54.40	56.74	32.52	24.48	35.56	20.19	18.23^{***}	< .001	0.10
Original d. (TRT)	42.03	39.67	32.19	19.90	42.09	24.98	6.70^{**}	.003	0.04
Clip d. (VE)	41.20	19.77	30.01	18.40	32.83	31.94	11.94^{***}	< .001	0.07
Clip d. (TRT)	40.18	21.39	31.60	18.68	34.83	25.11	7.93^{***}	< .001	0.05

Note. Effects of video speeds on the estimations of accuracy are presented. d. = duration; VE = verbal estimation; TRT = time reproduction task.
$^{**}p < .01$. $^{***}p < .001$.

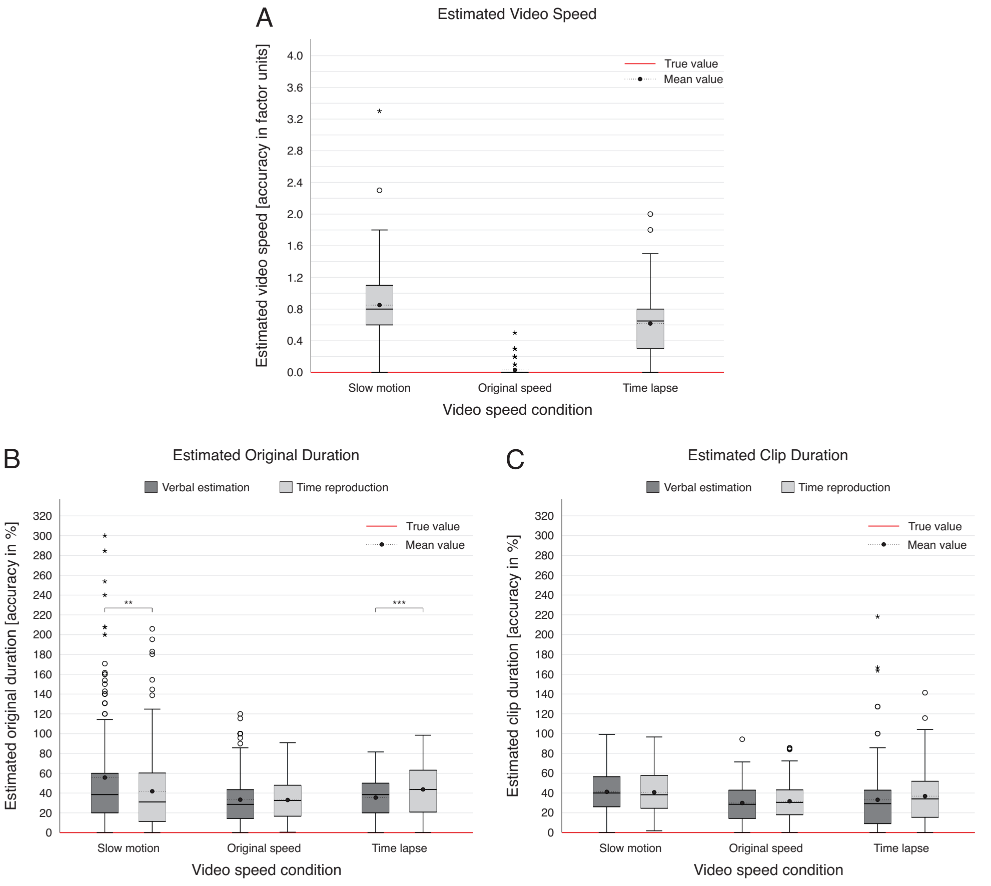

Fig. S1 Distribution of Accuracy in Duration and Speed Estimations Across Various Playback Speeds. Panel A illustrates the accuracy in video speed estimations, Panel B in original duration estimations, and Panel C in clip duration estimations, separated by estimation method (VE for verbal estimations, TRT for time reproduction tasks). The interquartile range is represented by the box, with the median as a line and the mean indicated by a black dot within it. Outliers are depicted as individual points and extreme values as stars. The true value for each condition is marked with a red line. Note that the sample size varies across conditions and can be determined from SI, Table S4. Significance brackets in Panels B and C denote statistically significant differences between VE and TRT. $^{**}p < .01$. $^{***}p < .001$.

Table S3 Post hoc Comparisons for Video Speed Effects on Speed and Duration Estimations for Accuracy

Variable	Comparison	M_{Diff}	*SE*	*p*	*n*	95% CI	*d*
Speed factor	SM vs. OS	0.80*	0.04	< .001	112	[0.71, 0.89]	1.98
	SM vs. TL	0.21*	0.04	< .001	112	[0.12, 0.31]	0.51
	OS vs. TL	-0.58*	0.04	< .001	112	[-0.67, -0.50]	-1.53
Orig. duration (VE)	SM vs. OS	21.88*	4.64	< .001	173	[10.66, 33.10]	0.36
	SM vs. TL	19.04*	4.46	< .001	173	[8.27, 29.82]	0.32
	OS vs. TL	-2.84	2.28	.644	173	[-8.34, 2.67]	-0.09
Orig. duration (TRT)	SM vs. OS	9.85*	3.52	.017	167	[1.34, 18.36]	0.22
	SM vs. TL	-0.06	3.49	1.00	167	[-8.50, 8.38]	-0.00
	OS vs. TL	-9.91*	2.14	< .001	167	[-15,09, -4.72]	-0.36
Clip duration (VE)	SM vs. OS	11.19*	1.37	< .001	171	[7.87, 14.51]	0.62
	SM vs. TL	8.37*	2.79	.009	171	[1.63, 15.11]	0.23
	OS vs. TL	-2.82	2.71	.899	171	[-9.39, 3.74]	-0.08
Clip duration (TRT)	SM vs. OS	8.58*	1.83	< .001	162	[4.15, 13.01]	0.37
	SM vs. TL	5.35	2.43	.088	162	[-0.54, 11.24]	0.17
	OS vs. TL	-3.23	2.22	.444	162	[-8.59, 2.14]	-0.11

Note. Bonferroni-corrected post hoc tests for multiple comparisons are reported following the significant rmANOVA. SM = slow motion; OS = original speed; TL = time lapse; VE = verbal estimation; TRT = time reproduction task; CI = confidence interval.
*$p < .05$.

Table S4 Accuracy Analysis: Descriptive Statistics and One-Sample *t* Test Results for Duration and Speed Estimations

Variable	Deviations		Δ	*t*	*df*	*p*	*d*
	M	*SD*	[factor units]				
Video speed							
Slow motion	0.85	0.45	0.85	22.97	145	< .001	1.90
Original speed	0.03	0.08	0.03	4.43	139	< .001	0.37
Time lapse	0.62	0.37	0.51	20.78	155	< .001	1.66

Variable	Accuracy		Δ		*t*	*df*	*p*	*d*
	M	*SD*	[%]	[s]				
Original duration (VE)								
Slow motion	55.67	57.76	55.67	3.38	12.75	174	< .001	0.96
Original speed	33.29	25.38	33.29	2.05	17.36	174	< .001	1.31
Time lapse	35.40	20.14	35.40	2.23	23.19	173	< .001	1.76
Original duration (TRT)								
Slow motion	41.80	39.66	41.80	0.82	13.66	167	< .001	1.05
Original speed	33.00	20.55	33.00	2.03	21.12	172	< .001	1.61
Time lapse	43.68	25.93	43.68	2.72	22.41	176	< .001	1.69
Clip duration (VE)								
Slow motion	41.18	19.81	41.18	5.77	27.66	176	< .001	2.08
Original speed	29.91	18.46	29.91	1.86	21.31	172	< .001	1.62
Time lapse	33.16	32.00	33.16	0.88	13.66	173	< .001	1.04
Clip duration (TRT)								
Slow motion	40.78	21.76	40.78	5.7	24.37	168	< .001	1.87
Original speed	31.73	19.00	31.73	1.98	21.51	165	< .001	1.67
Time lapse	36.85	26.55	36.85	0.99	18.31	173	< .001	1.39

Note. The table presents the mean parameter values for accuracy for each dependent variable across different video speeds, detailing both percentage accuracy and accuracy in seconds relative to the true value. Additionally, the table includes results from *t* tests that compare the accuracy with the true value (i.e. 100% for duration accuracies and 0 for speed accuracy, respectively), i.e. testing the differences against zero. VE = verbal estimation; TRT = time reproduction task; plus sign (+) = overestimation; minus sign (-) = underestimation.

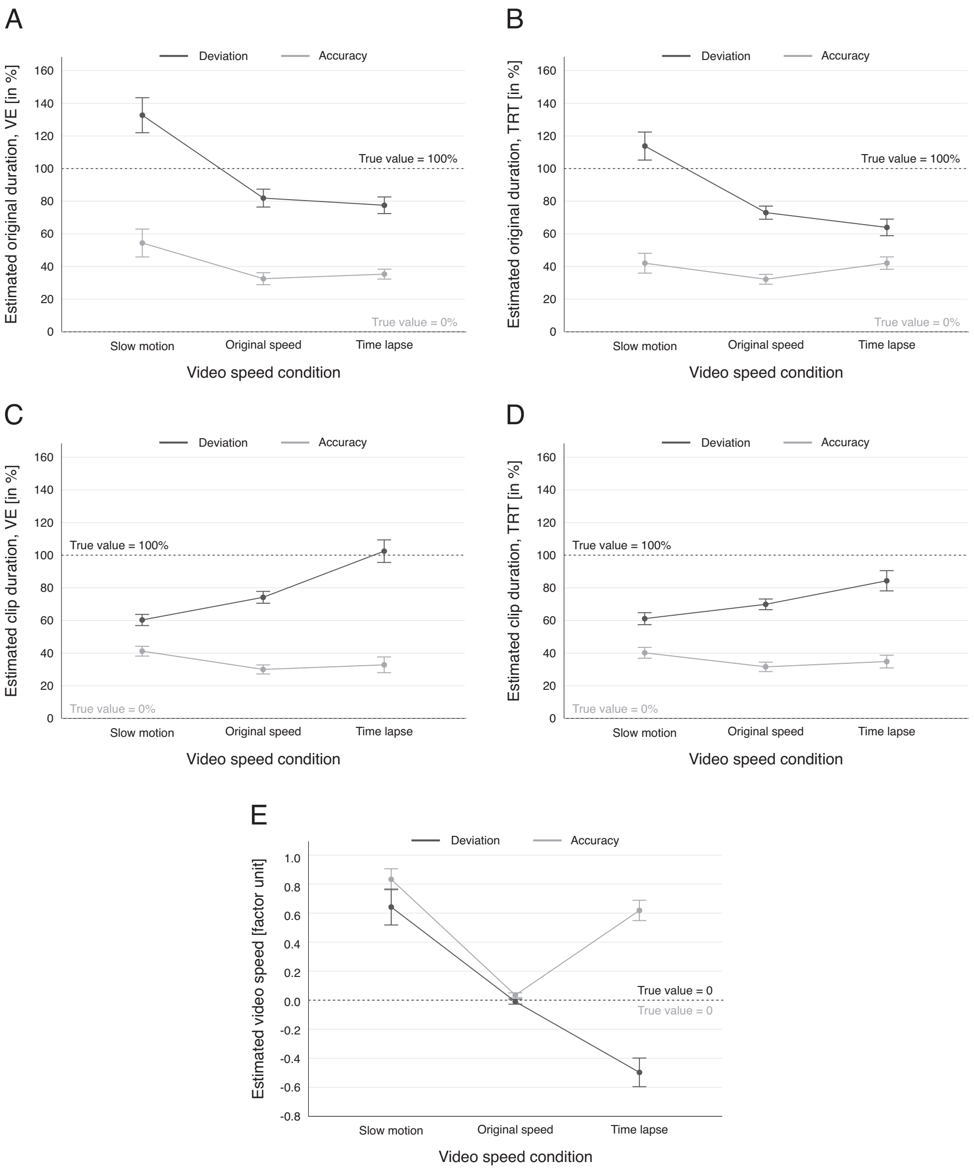

Fig. S2 Deviations and Accuracy of Video Speed and Duration Estimations Across Different Video Speed Conditions. Mean estimations for original and clip duration, as well as video speed across different playback speeds. Panels A and C show verbal estimates (VE); Panels B and D use a time reproduction task (TRT); Panel E presents estimates of video speed. Black lines mark deviations, grey lines show accuracy, and dotted lines serve as reference points with black at 100 indicating no deviation and grey at 0 indicating perfect accuracy. Error bars represent 95% confidence intervals.

B.2 Correlations

Table S5 Correlations for the Variables in Slow Motion

Variable	1	2	3	4	5
1. Original duration (VE)	-	.29**	-.35**	.19*	-.18*
2. Speed factor	.21**	-	-.10	.14	-.05
3. Clip duration (VE)	.48**	.06	-	-.11	.28**
4. Original duration (TRT)	.42**	.15	.23**	-	-.18*
5. Clip duration (TRT)	.18*	.08	.23**	.29**	-

Note. Pearson correlation coefficients are reported. The lower diagonal shows the correlations for the deviations, the upper diagonal the values for the accuracy variables.
$^{*}p < .05$. $^{**}p < .01$.

Table S6 Correlations for the Variables in Original Speed

Variable	1	2	3	4	5
1. Original duration (VE)	-	-.06	.32**	.09	.21**
2. Speed factor	.01	-	-.12	-.01	-.07
3. Clip duration (VE)	.56**	.03	-	.17*	.33**
4. Original duration (TRT)	.18*	.13	.18*	-	.44**
5. Clip duration (TRT)	.22**	.02	.36**	.54**	-

Note. Pearson correlation coefficients are reported. The lower diagonal shows the correlations for the deviations, the upper diagonal the values for the accuracy variables.
$^{*}p < .05$. $^{**}p < .01$.

Table S7 Correlations for the Variables in Time Lapse

Variable	1	2	3	4	5
1. Original duration (VE)	-	.07	.06	.18*	.08
2. Speed factor	-.08	-	-.11	.08	-.10
3. Clip duration (VE)	.42**	-.10	-	-.03	.31**
4. Original duration (TRT)	.33**	.06	.00	-	.20**
5. Clip duration (TRT)	.27**	.05	.16*	.33**	-

Note. Pearson correlation coefficients are reported. The lower diagonal shows the correlations for the deviations, the upper diagonal the values for the accuracy variables.
$^{*}p < .05$. $^{**}p < .01$.

B.3 Comparison of Measurement Methods

Table S8 Means, Standard Deviations, and Dependent *t* Test Statistics for Accuracy Variables

Variable	VE		TRT		*t*	*df*	*p*	95% CI	*d*
	M	*SD*	*M*	*SD*					
Original duration									
SM	55.80	58.77	41.62	39.71	2.85	166	.005	[4.36, 24.02]	0.22
OS	33.53	25.52	23.80	20.28	0.31	170	.759	[-3.97, 5.44]	0.02
TL	35.40	20.14	43.75	25.94	-3.70	173	< .001	[-12.80, -3.89]	-0.28
Clip duration									
SM	40.36	19.40	40.78	21.76	-0.22	168	.823	[-4.17, 3.33]	-0.02
OS	30.00	17.85	31.47	18.93	-0.89	163	.374	[-4.78, 1.80]	-0.07
TL	33.58	32.11	36.46	26.25	-1.09	170	.277	[-8.10, 2.34]	-0.08

Note. Mean accuracy of original and clip duration, verbal estimation vs. time reproduction task, across different video speed conditions are presented. SM = slow motion; OS = original speed; TL = time lapse; VE = verbal estimation; TRT = time reproduction task; CI = confidence interval.

C Analyses for the Five Key Variables Using Log-Transformed Data

In the main analysis, the ratio of the estimated value and the true value, multiplied by 100, was used to assess the deviation variables. However, this approach may introduces potential biases, as underestimations are bounded between 0% and 100%, while overestimations can exceed 100% without limit. This creates an imbalance where overestimates may disproportionately influence the average score. To mitigate this potential bias, one option is to apply a log transformation to the ratio of estimated to true values. This transformation generates an index centered on 0, with negative values indicating underestimation and positive values reflecting overestimation, thereby reducing the disproportionate impact of overestimations. After double-checking the findings in the main manuscript using log-transformed data, the overall pattern of results remained consistent with only a very few exceptions. Below, we present the results for the log-transformed data, including the rmANOVAs (Section C.1), correlations (Section C.2), and comparisons of measurement methods (Section C.3), with any differences in the significances highlighted in red.

C.1 Repeated Measures ANOVAs

All rmANOVAs remained significant, demonstrating the same significance patterns as the analysis with the deviation variables:

Significant differences in *original duration (VE)* deviations across video speed conditions were found, $F(1.93, 336.82) = 93.86$, $p < .001$, $\eta_p^2 = .35$ (large effect size).

Significant differences in *original duration (TRT)* deviations across video speed conditions were found, $F(1.75, 289.22) = 82.38$, $p < .001$, $\eta_p^2 = .33$ (large effect size).

Significant differences in *clip duration (VE)* deviations across video speed conditions were found, $F(1.76, 307.10) = 118.97$, $p < .001$, $\eta_p^2 = .41$ (large effect size).

Significant differences in *clip duration (TRT)* deviations across video speed conditions were found, $F(1.92, 313.23) = 29.26$, $p < .001$, $\eta_p^2 = .15$ (large effect size).

Table S9 Post hoc Comparisons for Video Speed Effects on Speed and Duration Estimations for Log-transformed Variables

Variable	Comparison	M_{Diff}	*SE*	*p*	95% CI
Speed factor	SM vs. OS	0.80*	0.04	< .001	[0.71, 0.89]
	SM vs. TL	0.21*	0.04	< .001	[0.12, 0.31]
	OS vs. TL	-0.58*	0.04	< .001	[-0.67, -0.50]
Original duration (VE)	SM vs. OS	0.19*	0.02	< .001	[0.15, 0.23]
	SM vs. TL	0.22*	0.02	< .001	[0.17, 0.26]
	OS vs. TL	0.03	0.02	.311	[-0.01, 0.07]
Original duration (TRT)	SM vs. OS	0.17*	0.02	< .001	[0.13, 0.21]
	SM vs. TL	0.25*	0.02	< .001	[0.20, 0.31]
	OS vs. TL	0.08*	0.02	< .001	[0.04, 0.13]
Clip duration (VE)	SM vs. OS	-0.11*	0.01	< .001	[-0.14, -0.08]
	SM vs. TL	-0.23*	0.02	< .001	[-0.28, -0.19]
	OS vs. TL	-0.13*	0.02	< .001	[-0.16, -0.09]
Clip duration (TRT)	SM vs. OS	-0.07*	0.02	< .001	[-0.11, -0.04]
	SM vs. TL	-0.13*	0.02	< .001	[-0.18, -0.09]
	OS vs. TL	-0.06*	0.02	.002	[-0.10, -0.02]

Note. Bonferroni-corrected post hoc tests for multiple comparisons are reported following the significant rmANOVA. This analysis showed no differences in the significance patterns compared to the main analysis with the deviation variables. SM = slow motion; OS = original speed; TL = time lapse; VE = verbal estimation; TRT = time reproduction task; CI = confidence interval.

*$p < .05$.

C.2 Correlations

Table S10 Correlations for the Variables in Slow Motion

Variable	1	2	3	4	5
1. Original duration (VE)	-				
2. Speed factor	.18*	-			
3. Clip duration (VE)	.48**	.07	-		
4. Original duration (TRT)	.50**	.15	.26**	-	
5. Clip duration (TRT)	.14	.10	.34**	.28**	-

Note. Pearson correlation coefficients are reported, showing correlations for log-transformed variables. This analysis showed no differences in the significance patterns compared to the main analysis with the deviation variables.
$^{*}p < .05.$ $^{**}p < .01.$

Table S11 Correlations for the Variables in Original Speed

Variable	1	2	3	4	5
1. Original duration (VE)	-		.		
2. Speed factor	-.06	-			
3. Clip duration (VE)	.56**	.01	-		
4. Original duration (TRT)	.04	.16	-.05	-	
5. Clip duration (TRT)	.27**	.02	.30**	.51**	-

Note. Pearson correlation coefficients are reported, showing correlations for log-transformed variables. Relevant differences in the significance patterns between the deviation variables and log-transformed variables are highlighted in red.
$^{*}p < .05.$ $^{**}p < .01.$

Table S12 Correlations for the Variables in Time Lapse

Variable	1	2	3	4	5
1. Original duration (VE)	-				
2. Speed factor	-.03	-			
3. Clip duration (VE)	.48**	-.19*	-		
4. Original duration (TRT)	.26**	.04	-.07	-	
5. Clip duration (TRT)	.13	.01	.09	.48**	-

Note. Pearson correlation coefficients are reported, showing correlations for log-transformed variables. Relevant differences in the significance patterns between the deviation variables and log-transformed variables are highlighted in red.
$^{*}p < .05.$ $^{**}p < .01.$

C.3 Comparison of Measurement Methods

Table S13 Means, Standard Deviations, and Dependent t Test Statistics for Log-transformed Variables

Variable	VE		TRT		t	df	p	95% CI	d
	M	SD	M	SD					
Original duration									
SM	0.07	0.23	0.01	0.23	3.68	167	$< .001$	[0.03, 0.10]	0.28
OS	-0.11	0.20	-0.17	0.18	3.13	171	.002	[0.02, 0.10]	0.24
TL	-0.13	0.21	-0.27	0.28	6.00	173	$< .001$	[0.09, 0.18]	0.46
Clip duration									
SM	-0.24	0.16	-0.25	0.19	0.34	166	.737	[-0.03, 0.04]	-0.03
OS	-0.14	0.15	-0.17	0.15	2.05	165	.042	[0.00, 0.06]	0.16
TL	-0.02	0.21	-0.13	0.24	5.08	173	$< .001$	[0.07, 0.16]	0.39

Note. Log-transformed values of original and clip duration, verbal estimation vs. time reproduction task, across different video speed conditions are presented. Relevant differences in the significance patterns between the deviation variables and log-transformed variables are highlighted in red. SM = slow motion; OS = original speed; TL = time lapse; VE = verbal estimation; TRT = time reproduction task; CI = confidence interval.

D Clip Independence

The results were validated by examining whether the mean ratings, calculated for each video speed condition across all three video clips, were influenced by the specific video clip content using a one-way analysis of variance (ANOVA) with the video clips (A, B, C) as between-subject factor. Bonferroni-corrected post hoc tests were applied for all conducted ratings. In case Levene's test indicated non-homogeneity of variance, a Welch ANOVA was conducted.

One important goal was to ensure that estimations remained independent of clip content, despite using different video clips, by generating and selecting similar clip designs and content. To further investigate this independence, a one-way ANOVA was conducted separately for each video speed to compare *video speed*, *original duration*, and *clip duration* estimations, with the video clip (video A: dice, video B: teapot, video C: match) as a between-subject factor.

No significant differences were found in the estimates based on the three different types of clip content. Only small but significant effect was found in the *video speed* estimations in slow motion, $F(2, 85.02) = 6.07$, $p = .003$, $\eta_p^2 = .05$, representing a small effect size. Pairwise comparisons revealed a significant difference between the estimations of video A in slow motion ($M = 0.48$ factor units, $SD = 0.73$), which were 0.38 factor units lower than those of video C ($M = 0.86$ factor units, $SD = 0.37$), $M_{\text{Diff}} = -0.38$, $p = .027$, 95% CI [-0.72, -0.03].

E Additional Analyses

For these further analyses involving the exploratory variables collected, Pearson's r was utilized for the age variable. For all other exploratory ratings, Spearman's rank-order correlation (ρ) was employed. Two-sample t tests were conducted for exploring gender differences.

E.1 Influence of Demographic Variables

In the following presentation of the results, the exploratory analyses of the demographic variables examine their relationship with the main variables.

Age

Deviations. As indicated by Pearson's correlation coefficient among all investigated deviation variables, only two small but significant negative correlations were found. Notably, this was observed for the *original duration (VE)* in slow motion, $r(173) =$ -.16, $p = .034$, and for the *video speed* in slow motion, $r(144) =$ -.24, $p = .004$. Essentially, with increasing age, participants tended to estimate the *original duration* in slow motion as shorter and perceive the *video speed* as slower, which implies estimating a higher slow motion factor.

Accuracy. No correlations were found among the accuracy variables with age.

Gender

Deviations. To quantify the extent of the differences in perception between genders (level 0 was assigned to female, and level 1 was assigned to male), an independent samples t test was conducted. The comparison between gender estimates showed a small but significant difference in the perception of *video speed* in slow motion, $t(139.60) =$ -2.58, $p = .011$, $d =$ -0.39. Men ($M = 0.82$ factor units, $SD = 0.49$) estimated *video speed* in slow motion 0.28 factor units higher (faster) than women ($M = 0.54$ factor units, $SD = 0.80$), 95% CI [-0.49, -0.06].

Accuracy. To quantify the extent of the differences in perception between genders, an independent samples t test was conducted. The comparison between gender estimates showed that men ($M = 26.51\%$, $SD = 16.92$) estimated the *original duration (VE)* in original speed 10.59% more accurately than women ($M = 37.11\%$, $SD = 28.44$), $t(172.42) = 3.09$, $p = .002$. $d = 0.43$, 95% CI [3.82, 17.36]. Additionally, in estimating *clip duration (TRT)* in original speed, men's estimations were found to be 6.47% more accurate, $t(164) = 2.11$, $p = .036$, $d = 0.38$, [0.42, 12.51], indicating a small effect size.

Discussion: Impact of Age and Gender on Perceptual Judgments

Age. In the study, with participants ranging in age from 19 to 87 years, age-related correlations with the main variables were significant only in the slow motion condition, particularly evident in the estimation of *original duration (VE)* and *video speed.* No substantial age correlation emerged in other duration or speed estimations. The findings showed that with increasing age, participants tended to provide lower estimates for both *original duration (VE)* and *video speed* in slow motion, which could

indicate a potentially less pronounced overestimation bias among older age groups. This tendency of older adults to underestimate video speed in general aligns with the findings of Rossi, Montanaro, and de'Sperati (2018) and de'Sperati, Granato, and Moretti (2021), with the former attributing it to motor slowing, and extends these previous observations by an analogous relation between age and original duration in slow motion. Additionally, the observation of duration underestimation is consistent with research suggesting that time perception tends to accelerate with age, possibly due to factors like working memory constraints and slower processing speeds (Craik & Hay, 1999; Perbal, Droit-Volet, Isingrini, & Pouthas, 2002) (note that, any potential associations regarding video speed could hence possibly also be driven by differences in duration experience, especially since the different video speeds necessarily led to different clip lengths).

Gender. Again, a correlation between gender and the main variables emerged solely in the slow motion condition. There was a significant correlation between male gender and increased estimation of *video speed* in slow motion, with men perceiving *video speed* as faster by an average of 0.28 factor units compared to female participants. However, it is important to note that a direct link between male gender and the overestimation bias in video speed perception cannot be conclusively drawn, but this finding provides an initial indication that gender may be linked to video speed perception, especially in slow motion. Contrary to general time perception research (Block, Hancock, & Zakay, 2000; Carlson & Feinberg, 1970; Hancock & Rausch, 2010), no gender differences were found in *original duration* or *clip duration* perception in this study. The absence of gender differences, as opposed to the significant gender association found in slow motion *video speed* perception, could be indicative of interaction effects that are context-specific and may suggest that gender impacts on time perception may be influenced by variables such as age, familiarity with altered video speeds, or confidence in ratings, suggesting detailed investigations for future research.

E.2 Counting

In order to determine the impact of counting, the participants were asked: "Did you mentally count seconds for your estimations?". This question aimed to assess counting during the viewing of a clip and during the TRT. Answers were categorized as *no*, *sometimes*, *yes*, or *I do not remember*. This item was intended to examine the effects of counting on estimations, a subject of ongoing research (Grondin, Ouellet, & Roussel, 2004; Rattat & Droit-Volet, 2012; Thönes & Hecht, 2017).

In assessing whether participants mentally counted seconds while watching a video and during a TRT, with response options coded as 1 (*no*), 2 (*sometimes*), 3 (*yes*), it was found that during video viewing 85 individuals (48%) responded with *yes*, 68 (38%) with *sometimes*, and 23 (13%) with *no* ($M = 2.27$, $SD = 0.69$). In the TRT, 95 participants (54%) counted, 55 (31%) did so sometimes, and 26 (15%) did not count ($M = 2.18$, $SD = 0.67$). One participant in each category could not recall.

Deviations. When correlating the counting behavior while watching a video and during a TRT with the deviations, a significant positive correlation was found when counting during video viewing for *original duration (VE)* in original speed, $r_s(172) =$

.17, p = .025. For *clip duration (TRT)* in slow motion, a significant positive correlation was found both when counting during video viewing, $r_s(166) = .21$, p = .006, and during TRT, $r_s(166) = .19$, p = .014, and also in time lapse, $r_s(171) = .16$, p = .040, with both correlations indicating a small effect size. The findings indicate that increased counting behavior is associated with higher estimates.

Accuracy. The accuracy of *clip duration (VE)* in original speed was significantly negatively correlated with counting during video viewing, $r_s(170) = -.17$, p = .029, and *clip duration (TRT)* in slow motion, $r_s(166) = -.22$, p = .003. Similar negative correlations were observed when counting during the *TRT* for the latter estimations, $r_s(166) = -.18$, p = .019, indicating that increased counting during video viewing or the TRT led to more accurate estimations on the respective variables, with both correlations indicating a small effect size.

E.3 Memory

To explore the potential influence of earlier responses on subsequent estimations (Gotts, Chow, & Martin, 2012; Li, Wang, & Zaidel, 2023; Menon & Raghubir, 2003; Tversky & Kahneman, 1973; Wehrman, Wearden, & Sowman, 2020), participants were asked: "Did you remember your previous estimations/answers for videos with the same clip content during the experiment?". Participants' ability to recall these responses was measured on a 5-point scale, ranging from *not at all* to *always*.

Participants' ability to recall their previous estimations of clips with identical content, measured on a 5-point scale (1 = *not at all*, 5 = *always*), resulted in an average recall score of M = 2.91 (SD = 0.97). Specifically, 12 participants (7%) responded *not at all*, 45 participants (25%) *hardly*, 77 participants (44%) *partly*, 33 participants (19%) *often*, and 10 participants (6%) *always*.

Deviations. A Spearman correlation revealed that recall was significantly positively associated with estimates of *original duration (VE)* in slow motion, $r_s(173) = .21$, p = .006, and in the TRT, $r_s(166) = .16$, p = .034. *Clip duration (VE)* in time lapse also showed a significant positive correlation with recall, $r_s(172) = .19$, p = .012. The findings suggest that participants who could better recall their previous estimates/answers regarding videos with the same clip content tended to provide higher estimates.

Accuracy. No correlations were found between estimation accuracy and recall abilities.

E.4 Ease and Confidence of Estimations

Participants' ease and confidence in making estimations for each dependent variable were assessed using two items: "How easy were the following estimations for you?" and "How confident were you in your estimations?". Responses were rated on a 5-point scale, from *very easy/very confident* to *not easy at all/not confident at all*, to evaluate their potential influence on the estimations and response patterns (Ais, Zylberberg, Barttfeld, & Sigman, 2016; Bruno, Sudkamp, & Souto, 2023; Chehibi, Ferchichi, Farah, & Hadjali, 2022; Samaha, Switzky, & Postle, 2019).

Confidence of Estimations. In assessing the confidence in their estimations on a scale from 1 (*very easy*) to 5 (*very difficult*), the average responses for estimating the *original duration (VE)* (M = 3.24, SD = 0.83) were approximately the same for *original duration (TRT)* (M = 3.30, SD = 0.83). The participants were somewhat more confident in estimating the *clip duration (VE)* (M = 2.76, SD = 0.85). For *clip duration (TRT)* (M = 3.02, SD = 0.85), their perceived confidence was just as high as for the *video speed* (M = 3.02, SD = 0.86).

Deviations. The correlation analysis revealed a small significant negative correlation between participants' confidence and the deviations in estimating the *original duration (VE)*, $r_s(173)$ = -.22, p = .004. The same negative correlation was observed with the *TRT*, $r_s(166)$ = -.24, p = .002, indicating that higher confidence levels tended to provide lower estimations.

Accuracy. Furthermore, significant positive correlations were found between participants' confidence in their estimations of *video speed* in original speed, $r_s(138)$ = .23, p = .006, and with their estimations of *clip duration (TRT)* in time lapse, $r_s(172)$ = .17, p = .027, implying that participants offer lower estimations when they expressed greater confidence.

Ease of Estimations. In assessing the perceived ease of their estimations on a scale from 1 (*very easy*) to 5 (*very difficult*), the average responses for estimating the *original duration (VE)* (M = 3.19, SD = 0.82) were approximately the same for the TRT (M = 3.13, SD = 0.87). Estimating the *clip duration* was somewhat easier for participants, both through VE (M = 2.57, SD = 0.83) and via TRT (M = 2.79, SD = 0.81). The perceived ease of estimating the *video speed* was intermediate, with M = 2.88 (SD = 0.79).

Deviations. A Spearman correlation showed that perceived ease was slightly but significantly negatively correlated with the deviations in estimating *original duration (VE)* in slow motion, $r(173)$ = -.15, p = .044, *clip duration (VE)* in slow motion, $r_s(175)$ = -.20, p = .008, and *original duration (TRT)* in slow motion, $r_s(168)$ = -.16, p = .040, indicating that a greater sense of ease led to higher estimations.

Accuracy. Accuracy was found to have a small positive correlation with the perceived ease of estimating *clip duration (VE)* in slow motion, $r_s(175)$ = .19, p = .011, and *clip duration (TRT)* in time lapse, $r_s(172)$ = .24, p = .002, suggesting that a higher perceived ease was associated with more accurate estimations.

E.5 Decimal Digits of Estimations

In light of research indicating preferences for rounding to numbers ending in "0" or "5" in VEs (Asaoka & Watanabe, 2015; Spencer, 1921; Yerkes & Urban, 1906), the frequency of these numerical endings was analyzed for *video speed*, *original duration (VE)*, and *clip duration (VE)* to identify potential effects.

The focus of the analysis was on the decimal places provided by participants in their estimates. In the following, the percentages refer to the smallest percentage of the respective variables across the slow motion, original speed and time lapse conditions).

In VE, it was consistently observed that participants showed a preference for numbers ending in "0", indicating a tendency to report whole numbers without a decimal place for both *original duration (VE)* (¿ 76%) and *clip duration (VE)* (> 64%). When a decimal place was specified, the most frequent choice was "5", occurring in > 7% of *original duration (VE)* estimates and > 13% of *clip duration (VE)* estimates. However, in original speed condition, "0" emerged as the predominant choice (75%), followed by "2" (11%) and "5" (5%). Although decimal digits varied numerically from "0" to "9" in the estimates under all conditions, the *video speed* estimates in original speed showed a remarkable cluster effect only within the range "0" to "5".

Regarding these results it can be discussed that in this study VE required manually inputting a numerical rating for the speed factor within the chosen speed option, restricted to positive numbers above 1.0 for both slow motion and time lapse. This method allowed for an unrestricted scale, free from upper or lower bounds, unlike percentage scales which are capped at zero on the lower end. However, it is known that numerical entries, as also seen in the previously mentioned VEs of duration estimations, can be biased. An interesting finding in this study was that, across all speed conditions in duration estimation, and in video speed perception estimations in slow motion and time lapse, decimal digits varied numerically from "0" to "9" with the preferred digits of "0" followed by "5", but the *video speed* estimates in original duration showed a remarkable clustering effect within the range between "0" and "5", preferring endings with "0" followed by "2". For validation purposes, especially in the context of speed measurement methods, it would be beneficial to consider additional response options like sliders or Likert scales. The application of different psychophysical methods, each with its own advantages and disadvantages, such as adjustment tasks (which would require multiple viewings and significantly longer videos than those used in the present study, to allow also for adjustment of time lapse videos) or more perceptually-oriented staircase methods, both used by Rossi et al. (2018), could also be potential approaches for future research.

References

Ais, J., Zylberberg, A., Barttfeld, P., Sigman, M. (2016). Individual consistency in the accuracy and distribution of confidence judgments. *Cognition*, *146*, 377–386, https://doi.org/10.1016/j.cognition.2015.10.006

Asaoka, R., & Watanabe, Y. (2015). Differences between measuring methods of time estimation. *Tohoku Psychologica Folia*, *74*, 1–12,

Block, R.A., Hancock, P.A., Zakay, D. (2000). Sex differences in duration judgments: A meta-analytic review. *Memory & Cognition*, *28*(8), 1333–1346, https://doi.org/10.3758/BF03211834

Bruno, A., Sudkamp, J., Souto, D. (2023). A metacognitive approach to the study of motion-induced duration biases reveals inter-individual differences in forming confidence judgments. *Journal of Vision*, *23*(3), Article 15, https://doi.org/10.1167/jov.23.3.15

Carlson, V.R., & Feinberg, I. (1970). Time judgment as a function of method, practice, and sex. *Journal of Experimental Psychology*, *85*(2), 171–180, https://doi.org/10.1037/h0029504

Chehibi, M., Ferchichi, A., Farah, I.R., Hadjali, A. (2022). Managing temporal uncertainty - A short review. *2022 2nd International Conference of Smart Systems and Emerging Technologies (SMARTTECH)* (pp. 38–43).

Craik, F.I.M., & Hay, J.F. (1999). Aging and judgments of duration: Effects of task complexity and method of estimation. *Perception & Psychophysics*, *61*(3), 549–560, https://doi.org/10.3758/BF03211972

de'Sperati, C., Granato, M., Moretti, M. (2021). If you are old, videos look slow. The paradoxical effect of age-related motor decline on the kinematic interpretation of visual scenes. *Frontiers in Human Neuroscience*, *15*, Article 783090, https://doi.org/10.3389/fnhum.2021.783090

Gotts, S.J., Chow, C.C., Martin, A. (2012). Repetition priming and repetition suppression: A case for enhanced efficiency through neural synchronization. *Cognitive Neuroscience*, *3*(3-4), 227–237, https://doi.org/10.1080/17588928.2012.670617

Grondin, S., Ouellet, B., Roussel, M.-E. (2004). Benefits and limits of explicit counting for discriminating temporal intervals. *Canadian Journal of Experimental Psychology*, *58*(1), 1–12, https://doi.org/10.1037/h0087436

Hancock, P.A., & Rausch, R. (2010). The effects of sex, age, and interval duration on the perception of time. *Acta Psychologica*, *133*(2), 170–179, https://doi.org/10.1016/j.actpsy.2009.11.005

Hüttner, N., Sperl, L., Schroeger, A. (2023). Slow motion bias: Exploring the relation between time overestimation and increased perceived intentionality. *Perception*, *52*(2), 77–96, https://doi.org/10.1177/03010066221139943

Li, B., Wang, B., Zaidel, A. (2023). Modality-specific sensory and decisional carryover effects in duration perception. *BMC Biology*, *21*, Article 48, https://doi.org/10.1186/s12915-023-01547-9

Maaß, S.C., Riemer, M., Wolbers, T., van Rijn, H. (2019). Timing deficiencies in amnestic Mild Cognitive Impairment: Disentangling clock and memory processes. *Behavioural Brain Research*, *373*, Article 112110, https://doi.org/10.1016/j.bbr.2019.112110

Matthews, W.J. (2011). How do changes in speed affect the perception of duration? *Journal of Experimental Psychology Human Perception & Performance*, *37*(5), 1617–1627, https://doi.org/10.1037/a0022193

Menon, G., & Raghubir, P. (2003). Ease-of-retrieval as an automatic input in judgments: A mere-accessibility framework? *Journal of Consumer Research*, *30*(2), 230–243, https://doi.org/10.1086/376804

Perbal, S., Droit-Volet, S., Isingrini, M., Pouthas, V. (2002). Relationships between age-related changes in time estimation and age-related changes in processing speed, attention, and memory. *Aging, Neuropsychology, and Cognition*, *9*(3), 201–216, https://doi.org/10.1076/anec.9.3.201.9609

Rattat, A.-C., & Droit-Volet, S. (2012). What is the best and easiest method of preventing counting in different temporal tasks? *Behavior Research Methods*, *44*(1), 67–80, https://doi.org/10.3758/s13428-011-0135-3

Rossi, F., Montanaro, E., de'Sperati, C. (2018). Speed biases with real-life video clips. *Frontiers in Integrative Neuroscience*, *12*, Article 11, https://doi.org/10.3389/fnint.2018.00011

Samaha, J., Switzky, M., Postle, B.R. (2019). Confidence boosts serial dependence in orientation estimation. *Journal of Vision*, *19*(4), Article 25, https://doi.org/10.1167/19.4.25

Spencer, L.T. (1921). An experiment in time estimation using different interpolations. *The American Journal of Psychology*, *32*(4), 557–562, https://doi.org/10.2307/1413775

Sperl, L., Hüttner, N., Schroeger, A. (2021). Why do actions in slow motion appear to last longer? On the effect of video speed information. *Perception*, *50*(1), 69–79, https://doi.org/10.1177/0301006620982212

Thönes, S., & Hecht, H. (2017). Counting does not improve the accuracy of long time productions. *Attention, Perception, & Psychophysics*, *79*(8), 2576–2589, https://doi.org/10.3758/s13414-017-1407-5

Tversky, A., & Kahneman, D. (1973). Availability: A heuristic for judging frequency and probability. *Cognitive Psychology*, *5*(2), 207–232, https://doi.org/10.1016/0010-0285(73)90033-9

Wehrman, J.J., Wearden, J., Sowman, P. (2020). Decisional carryover effects in interval timing: Evidence of a generalized response bias. *Attention, Perception, & Psychophysics*, *82*(4), 2147–2164, https://doi.org/10.3758/s13414-019-01922-1

Yerkes, R.M., & Urban, F.M. (1906). Time estimation in its relation to sex, age, and physiological rhythms. *Harvard Psychological Studies*, *2*, 405–430,
